# Aromatase and glycosyl transferase inhibiting acridone alkaloids from fruits of Cameroonian *Zanthoxylum* species

**DOI:** 10.1186/1752-153X-7-125

**Published:** 2013-07-18

**Authors:** Vyry NA Wouatsa, Laxminarain Misra, Shiv Kumar, Om Prakash, Feroz Khan, Francois Tchoumbougnang, R Kumar Venkatesh

**Affiliations:** 1 CSIR- Central Institute of Medicinal and Aromatic Plants, Lucknow 226015, India; 2 Institute of Fisheries Sciences, University of Douala, Douala 24157, Cameroon; 3 Babasaheb Bhimrao Ambedkar University, Lucknow 226025, India; 4 Chemical Sciences Division, CSIR- Central Institute of Medicinal and Aromatic Plants, Kuckrail Picnic Spot Road, P.O. CIMAP, Lucknow 226015, India

**Keywords:** Rutaceae, *Zanthoxylum zanthoxyloides*, *Z. leprieurii*, Zanthacridones, Structure elucidation, Antibacterial, Cytotoxic activities, Molecular modeling, QSAR studies

## Abstract

**Background:**

*Zanthoxylum zanthoxyloides* and *Z. leprieurii* fruits are commonly used in traditional system of medicine for diarrhea, pain, wound healing, etc. in Cameroon, Africa. *Z. leprieurii* fruits have been chemically studied for its bioactive compounds whereas the investigation on *Z. zanthoxyloides* fruits is lacking.

**Results:**

After a detailed chemical analysis of the fruits of *Z. leprieurii* and *Z. zanthoxyloides*, a series of new acridone alkaloids, namely, 3-hydroxy-1,5,6-trimethoxy-9-acridone (**1**), 1,6-dihydroxy-3-methoxy-9-acridone (**2**), 3,4,5,7-tetrahydroxy-1-methoxy-10-methyl-9-acridone (**3**), 4-methoxyzanthacridone (**8**), 4-hydroxyzanthacridone (**9**), 4-hydroxyzanthacridone oxide (2,4’) (**10**) have been isolated. The known acridones which have been characterized are, helebelicine A (**4**), 1-hydroxy-3-methoxy-10-methyl-9-acridone (**5**)*,* 1,3-dihydroxy-4-methoxy-10-methyl-9-acridone (**6**) and tegerrardin A (**7**). The i*n vitro* antibacterial and cytotoxic screening of these acridones reveal that compound **3** has a moderate antibacterial activity (MIC 125 μg/mL) against *Micrococcus luteus* and *Pseudomonas aeruginosa* while compound **1** shows a moderate cytotoxic effect (IC_50_ of 86 μM) against WRL-68 (liver cancer cell line). Furthermore, the molecular modeling of these acridones predicted the structural basis for their mode of action and binding affinity for aromatase, quinone reductase and WAAG, a glycosyltransferase involved in bacterial lipopolysaccharide synthesis. Computational approaches, quantitative SAR and modeling studies predicted that acridones **1, 2, 3, 4, 9** and **10** were the inhibitors of glycosyltransferase while **1**, **8, 9** and **10,** the inhibitors of aromatase.

**Conclusions:**

A total of 10 acridones have been isolated out of which 6 are new (**1**, **2**, **3**, **8**, **9** and **10**). Alkaloids **8**, **9** and **10**, having novel tetracyclic acridone structure with new carbon skeleton, have now been named as zanthacridone. The quantitative SAR and molecular modeling studies suggested that the compounds **1**, **9** and **10** are inhibitors of both aromatase and glycosyltransferase.

## Background

The pantropical genus *Zanthoxylum* (Rutaceae) is represented by 35 species in Africa [1]. Among them, *Zanthoxylum zanthoxyloides* (Lam.) Zepern. & Timler and *Zanthoxylum leprieurii* Guill. et Perr are found in Cameroon and are well reputed for their ethnomedicinal properties. *Z. zanthoxyloides,* formerly known as *Fagara zanthoxyloides,* a popular African medicinal plant, occurs abundantly in savanna and dry forest vegetations whereas *Z. leprieurii*, is a medium tree found in rain forests and savanna woodland, from sea-level up to the altitude of 2000 m [1,2]. In Cameroon, the leaves, stems and roots of these plants are used in the treatment of gonococci, urinary infections and dysentery [3]. In addition, the fruit infusion of *Z. leprieurii* is shown to relegate the symptoms of sickle cell anemia [2]. The aqueous ethanolic extracts of the leaves, roots and stem bark of *Z. leprieurii* have demonstrated moderate antifungal activity while the chloroform extract of the fruits showed moderate cytotoxicity with the brine-shrimp assay [4,5]. Recent reports on the dried fruits of *Z. leprieurii* from Cameroon described the isolation of acridone alkaloids that exhibited antiplasmodial activity and *in vitro* cytotoxicity [5,6]. Its roots and stem bark have yielded alkaloids (benzophenanthridines, acridone, aporphine), aliphatic amides and lignans [5,7,8]. Several studies on *Z. zanthoxyloides* have established its broad spectrum of biological activities, including sickle cell anemia [9-11]. The antisickling divanylloylquinic acids as well as the antifungal and antioxidant isobutylamide and benzophenanthridine alkaloids have been reported from the roots [12-14]. From its fruits, the components of essential oils were also characterized [15,16]. Besides the studies on its essential oil, no work has been carried out on the constituents of its non volatile extract. A recent study has revealed that the alcoholic extract of the fruits of this plant possessed *in vitro* cytotoxic activity against MCF-7, without identifying any bioactive constituents [17]. This prompted us to undertake a comparative study on the chemical constituents of the fruits of *Z. zanthoxyloides* and *Z. leprieurii* to identify their bioactive compounds.

In continuation of our work on the chemistry of medicinal and aromatic plants [18-22], we have investigated the fruits of two *Zanthoxylum* species collected from Cameroon, Africa and recently reported their bioactive non-alkaloidal phytoconstituents [23,24]. In the present paper, we describe the isolation and structure elucidation of several new acridone alkaloids, some of them with novel structures, from *Z. zanthoxyloides* and *Z. leprieurii* exhibiting antibacterial and cytotoxic activities. In order to explore the possible mechanism of these activities, we have also performed quantitative SAR of these acridones along with their molecular docking experiments to identify their putative biological targets.

## Results and discussion

### Isolation and identification of acridones

**Figure 1:**
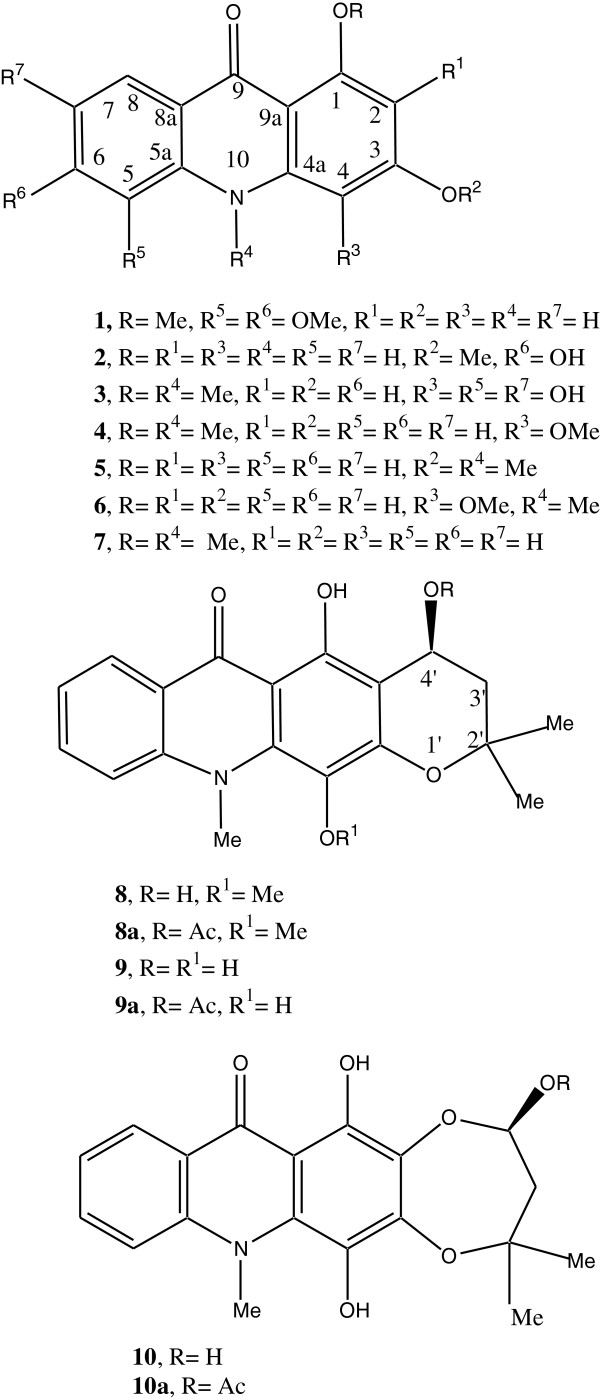
Structures of acridones 1- 10.

From the MeOH extract of the fruits of *Z. zanthoxyloides,* 6 new acridone alkaloids viz., 3-hydroxy-1,5,6-trimethoxy-9-acridone (**1**), 1,6-dihydroxy-3-methoxy-9-acridone (**2**), 3,4,5,7-tetrahydroxy-1-methoxy-10-methyl-9-acridone (**3**), 4-methoxyzanthacridone (**8**), 4-hydroxyzanthacridone (**9**), 4-hydroxyzanthacridone oxide (2,4’) (**10**) along with two known acridones, viz., helebelicine A (**4**) and 1-hydroxy-3-methoxy-10-methyl-9-acridone (**5**), have been isolated and identified (Figure 1). Compounds **8- 10** have a tetracyclic acridone carbon skeleton reported for the first time from *Zanthoxylum spp.* This novel acridone skeleton has, tentatively, been named as zanthacridone. From *Z. leprieuri* also*,* the new compound, 3-hydroxy-1,5,6-trimethoxy-9-acridone (**1**), was isolated along with three known acridones, namely, helebelicine A (**4**), 1,3-dihydroxy-4-methoxy-10-methyl-9-acridone (**6**) and tegerrardin A (**7**). All the compounds were Dragendorff positive and yellowish orange in color. The structures of the new compounds were elucidated mainly by UV, IR, NMR and MS spectroscopy (Additional file 1) and by comparison with the data already reported in the literature for acridone alkaloids [5,25-32].

Compound **1** in its UV spectrum showed bands at 220, 233, 252 and 320 nm while in IR the bands appeared at 3448, 2929, 1620 cm^-1^ which suggested the presence of an acridone skeleton. Its molecular formula was determined by HRMS as C_16_H_15_O_5_N with [M]^+^ at *m/z* 301.0944. On the basis of the molecular formula and the assumption of an acridone skeleton through comparison with the literature data for similar compounds [5], compound **1** must be a trimethoxy-hydroxy-acridone. In the unsubstituted acridone, the signals of the *peri*-protons H-1 and H-8 are found at δ 8.25, while the neighbors of N-10 are found at approximately δ 7.5. It follows that the doublet at δ 7.99 ppm belongs to H-8; the coupling constant indicated a proton (H-7) in *ortho*-position. As the signal of H-7 did not show further couplings, the positions C-5 and C-6 must be oxygenated. It was further substantiated as in the ^1^H^1^H COSY spectrum H-7 showed correlation only with H-8 and in HMBC it showed correlation with C-7-OMe at δ 61.9. Its ^1^H NMR spectrum (Table 1) showed a downfield singlet at δ 9.02 for C-3-OH along with two meta coupled doublets (J = 3.0 Hz) at δ 7.01 and 7.55 indicating that C-1 and C-3 were substituted. The presence of three singlets at δ 4.40, 4.10 and 4.01 each of 3H in ^1^H NMR spectrum and δ 61.9, 59.3 and 57.5 in its ^13^C NMR spectrum indicated that it contained 3 OMe groups. The HMBC spectrum showed correlations as reported earlier [5] for 1,3 oxygenated acridones (Experimental). Its ^13^C NMR spectrum also exhibited four CH signals at 113.5, 104.8, 118.3 and 162.3 along with four C-O-R singlets at δ 158.0, 152.6, 145.1 and 143.3. These sets of signals clearly implied that in acridone skeleton, A and C rings are oxidized at 4 positions having 3 methoxy and a hydroxy functional group. The absence of the singlet of a chelated hydroxyl group in the region of δ 10-15 in its ^1^H NMR spectrum indicated that the *peri* positions C-1 and C-8 were not hydroxylated. This was further reinforced by the presence of the strongly downfielded signals at δ 4.40 in its ^1^H NMR spectrum and at δ 158.0 and 61.9 for C-1-OMe in its ^13^C NMR spectrum. The meta coupled doublets at δ 7.01 and 7.55 (J = 3.0 Hz) for H-2 and H-4 indicated that C-3 is also oxidized which was supported by the typical signal for C-3 in acridones at δ 152.6 in its ^13^C NMR spectrum. The C-3-OH signal at δ 9.02 correlated with C-3 at δ 152.6 in its HMBC spectrum, clearly indicating the placement of phenol in A ring. The downfield signal at δ 7.99 in ^1^H NMR spectrum which is typical for H-8 showed ortho coupling (J = 8.0 Hz) with H-7, correlation in ^1^H^1^H COSY and no meta coupling, suggesting that two other methoxy groups are present at C-5 and C-6. The relatively strong upfield shifted C-4 signal at δ 104.8 in ^13^C NMR spectrum for aromatic carbon fitted well with the earlier report [5]. The rest of the signals of ^13^C NMR spectrum, when compared with the literature data, [5,30,33,34] supported that **1** is a 3-hydroxy-1,5,6-trimethoxy-9-acridone.

**Table 1:** ^**1**^**H NMR spectral data for 1-3, 8-10 (300 MHz, in CDCl**_**3**_**, δ in ppm)**

**Proton**	**1**	**2**	**3**	**8**	**9**	**10**
2	7.01(d,3.0)	6.58(d,3.0)	6.12(s)	-	-	-
4	7.55(d,3.0)	6.44(d,3.0)	-	-	-	-
5	-	7.39(d,3.0)	-	7.40(dd,8.0,3.0)	7.41(dd,8.0,3.0)	7.34(dd,8.0,3.0)
6	-	-	7.20(d,3.0)	7.61(ddd,8.0,8.0,3.0)	7.59(ddd,8.0,8.0,3.0)	7.55(ddd,8.0,8.0,3.0)
7	7.21(d,8.0)	7.07(dd,8.0,3.0)	-	7.29(ddd,8.0,8.0,3.0)	7.34(ddd,8.0,8.0,3.0)	7.34(ddd,8.0,8.0,3.0)
8	7.99(d,8.0)	7.89(d,8.0)	7.82(d,3.0)	7.82(dd,8.0,3.0)	8.40(dd, 8.0, 3.0)	8.39(dd,8.0,3.0)
1-OMe	4.40(s)	-	4.44(s)	-	-	-
3-OMe	-	3.93(s)	-	-	-	-
4-OMe	-	-	-	3.98(s)	-	-
5-OMe	4.10(s)	-	-	-	-	-
6-OMe	4.01(s)	-	-	-	-	-
N-Me	-	-	3.90(s)	3.77(s)	3.69(s)	3.70(s)
2’-Me	-	-	-	1.30(s)	1.33(s)	1.32(s)
				1.30(s)	1.42(s)	1.28(s)
3’	-	-	-	2.72(dd,13,10)	2.68(dd,13,3.5)	3.23(overlapping m)
				3.07(dd,13,3.5)	3.00(dd,13,3.5)	3.25(overlapping m)
4’	-	-	-	3.50(dd,10,3.5)	3.63(dd,10,3.5)	4.85(dd,10.0,10.0)
1-OH	-	12.12	-	15.05	15.00	14.92
3-OH	9.02	-	9.05*	-	-	-
4-OH	-	-	9.00*	-	9.05	9.10
5-OH	-	-	9.32*	-	-	-
6-OH	-	8.90	-	-	-	-
7-OH	-	-	8.92*	-	-	-

*Assignments interchangeable.

Compound **2** exhibited UV and IR signals as in case of **1**. Its HRMS showed [M]^+^ at *m/z* 257.0681 confirming C_14_H_11_O_4_N as its molecular formula. Its ^1^H NMR (Table 1) spectrum showed singlets at δ 12.12 and δ 8.90 for C-1-OH and C-6-OH, respectively, along with a relatively strong upfield singlet at δ 3.93 suggesting the presence of a methoxy group at C-3 in the acridone skeleton which was supported by the typical quartet at δ 56.4 in its ^13^C NMR spectrum. The ^13^C NMR spectrum (Table 2) further showed three downfield signals at δ 161.2, 162.4 and 144.0 suggesting 3 oxygenated carbons in A and C rings of acridone skeleton. The ^1^H NMR spectrum exhibited meta coupled doublets (J = 3.0 Hz) at δ 6.58 and 6.44 showing matching substitution in A ring as in case of compound **1**. The typical downfield doublet at δ 7.89 (J = 8.0 Hz) for H-8 in ^1^H NMR spectrum showed correlation with a double doublet (J = 8.0, 3.0 Hz) for H-7 at δ 7.07 which in turn correlated with the doublet (J = 3.0 Hz) for H-5 at δ 7.39 in its ^1^H^1^H COSY. These data suggested that the second hydroxyl group is attached at C-6. The relatively upfield shift of C-4 at δ 90.7 in ^13^C NMR spectrum for aromatic carbon has been observed in earlier study [5]. The rest of ^13^C NMR values (Experimental) accorded well with the structure for **2** as 1,6-dihydroxy-3-methoxy-9-acridone.

**Table 2:** ^**13**^**C NMR spectral data for 1-3, 8-10 (75 MHz, in CDCl**_**3**_**, δ in ppm)**

**Carbon**	**1**	**2**	**3**	**8**	**9**	**10**
1	158.0 (s)	161.2 (s)	160.0 (s)	162.1 (s)	160.0 (s)	159.3(s)
2	113.5 (d)	94.3 (d)	92.7 (d)	118.2 (s)	117.5 (s)	160.2 (s)
3	152.6 (s)	162.4 (s)	161.3 (s)	166.5 (s)	166.4 (s)	166.5 (s)
4	104.8 (d)	90.7 (d)	130.2 (s)	123.0 (s)	122.1 (s)	131.2 (s)
4a	143.3 (s)	140.7 (s)	141.2 (s)	139.3 (s)	139.0 (s)	124.8 (s)
5a	144.0 (s)	140.7 (s)	141.3 (s)	139.3 (s)	140.2 (s)	133.2 (s)
5	145.1 (s)	104.4 (d)	146.2 (s)	115.0 (d)	116.1 (d)	114.5 (d)
6	143.3 (s)	144.0 (s)	119.8 (d)	131.2 (d)	131.3 (d)	131.4 (d)
7	118.3 (d)	117.2 (d)	143.1 (s)	123.0 (d)	123.0 (d)	123.6 (d)
8	162.3 (d)	126.9 (d)	117.2 (d)	124.0 (d)	124.1 (d)	126.8 (d)
8a	115.6 (s)	120.4 (s)	121.5 (s)	121.5 (s)	121.6 (s)	122.1 (s)
9	178.2 (s)	180.8 (s)	181.1 (s)	177.2 (s)	178.3 (s)	181.6 (s)
9a	104.7 (s)	108.2 (s)	108.1 (s)	114.9 (s)	114.9 (s)	116.2 (s)
OMe	57.5 (q)	56.4 (q)	60.9 (q)	62.8 (q)	-	-
	59.3 (q)	-	-	-	-	-
	61.9 (q)	-	-	-	-	-
N-Me	-	-	34.3 (q)	30.5 (q)	31.5 (q)	31.7 (q)
2’	-	-	-	73.4 (s)	73.4 (s)	72.3 (s)
2’Me	-	-	-	24.4 (q)	25.0 (q)	24.7 (q)
				26.1 (q)	26.2 (q)	26.0 (q)
3’	-	-	-	28.1 (t)	28.2 (t)	28.1 (t)
4’	-	-	-	79.6 (d)	78.6 (d)	92.3 (d)

*The multiplicity of the signals obtained by DEPT 135.

Compound **3** had the UV and IR bands for acridone alkaloids as described in the case of **1**. Its HRMS showed [M]^+^ at *m/z* 303.0734 corresponding to C_15_H_13_O_6_N. Its ^1^H NMR spectrum showed four downfield singlets at δ 9.05, 9.32, 9.00 and 8.92 for phenolic protons. The downfield meta coupled doublet (J = 3.0 Hz) at δ 7.82 typical for H-8 in its ^1^H NMR spectrum, clearly suggested that the doublet (J = 3.0 Hz) at δ 7.20 corresponds to H-6. Its ^1^H NMR spectrum showed a typical downfield singlet at δ 4.44 for OMe at C-1 and δ 3.90 for N-Me which was supported by the quartets in ^13^C NMR spectrum at δ 60.9 and 34.3, respectively. A singlet at δ 6.12 in the ^1^H NMR spectrum further suggested that rest of the 3 carbons in A ring along with 2 in C ring are oxygenated which was strengthened by the presence of a total of five C-O-R signals in its ^13^C NMR spectrum at δ 160.0, 161.3, 130.2, 146.2 and 143.1 for C-1, C-3, C-4, C-5 and C-7, respectively. The relatively strong upfield shift of C-4 at δ 130.2 for oxygenated carbon with N-10 methylated acridones fits well with the published data [5]. These data (Tables 1 and 2) established that **3** is 3,4,5, 7-tetrahydroxy-1-methoxy-10-methyl-9-acridone.

Compound **8** showed UV and IR spectral values for acridone skeleton as described in the case of **1**. Its HRMS showed [M]^+^ at *m/z* 355.1416 for C_20_H_21_O_5_N. An OH (H-bonded) singlet appeared at δ 15.05 in its ^1^H NMR spectrum (Table 1) indicating the presence of a phenol at C-1. In its ^1^H NMR spectrum, two sets of double doublets (J = 8.0 Hz and 3.0 Hz) at δ 7.82 and 7.40 for H-8 and H-5 along with two sets of triple doublets (J = 8.0, 8.0, 3.0 Hz) at δ 7.61 and 7.29 for H-6 and H-7, were available. These signals showed respective correlations from H-5 to H-8 in the ^1^H^1^H COSY spectrum (Experimental) revealing that C ring is unsubstituted at C-5 to C-8 positions. This fact was justified by the doublets at δ 115.0, 131.2, 123.0 and 124.0 available in its ^13^C NMR spectrum (Table 2). Since no signals, other than these four for C ring, were seen in the typically unsubstituted aromatic region in ^1^H NMR spectrum of **8**, it was concluded that all the four free carbons in A ring were substituted. The downfield signals at δ 162.1, 166.5 and 123.0 in its ^13^C NMR spectrum implied that 3 carbons in A ring are oxygenated while 4th carbon is C-substituted. The relatively strong upfield shift of C-4 at δ 123.0 for oxygenated carbon with N-10 methylated acridones concurred well with previous report [5]. A set of additional signals in its ^1^H NMR spectrum at δ 3.50 (dd, J = 10, 3.5 Hz), δ 2.72 and 3.07 (dd each, J = 13.0, 10.0 Hz) and two methyl singlets (6H) at δ 1.30 designated the presence of a three carbon chain attached with A ring. The ^1^H^1^H COSY experiment showed a cyclic ring of -CH(OH)CH_2_C(O)(CH_3_)_2_- attached at C-2 and C-3. Although the oxidation at C-3 is biogenetically preferred in this class of compounds, the HMBC experiment established that C-2 is C-substituted while C-3 is O-substituted. In HMBC spectrum, the signal for 4’-H at δ 3.50 showed strong correlation with C-2 at δ 118.2. This type of cyclization is justified by the ^13^C NMR values at δ 79.6 (d), 28.1 (t), 73.4 (s) as well as 24.4 and 26.1 (q) along with C-2 and C-3 singlets at δ 118.2 and 166.5, respectively. In the NOESY spectrum of **8**, the C-4’-H at δ 3.50 showed a weak correlation with phenolic proton at C-1 at δ 15.05 and no correlation with C-2’-Me, suggesting that OH at C-4’ is β-oriented. The molecular model of **8** further reinforced this statement. Its HMQC and HMBC spectra also supported these observations (Experimental). The 3, 4-cyclization is normally preferred for a more stable linear structure of acridones. Recently, the linear tetracyclic acridones were reported from *Glycosmis parva* by Chansriniyom, et al. [25] having a double bond in pyran ring. Moreover, similar cyclization product was earlier reported from the synthesis of 1-hydroxy-1,2-dihydroisoacronycine by Magiatis et al. [35] but without oxidation at C-4. Acetylation of **8** yielded **8a** showing the downfield shifting of CH(OH) from δ 3.50 to δ 4.34 along with an additional signal at δ 2.10 for OAc in its ^1^H NMR spectrum. This observation clearly supported the presence of a CHOH group in this ring. Since the OMe appeared slightly upfield at δ 3.98 in ^1^H NMR, it was placed at C-4 and not at C-1, obviously indicating that C-1 is substituted by a hydroxy group. The typical signals at δ 3.77 (s) in ^1^H NMR and δ 30.5 (quartet) in ^13^C NMR spectra justified the presence of N-Me. The present tetracyclic skeleton was, tentatively, named as zanthacridone and the data described as above supported the structure of **8** as 4-methoxyzanthacridone.

Compound **9** had almost similar spectral data except the methoxy signal in ^1^H NMR and ^13^C NMR spectra (Tables 1 and 2) which clearly suggested that C-4 is now substituted with a hydroxy group rather than a methoxy, as was in case of compound **8**. In its HRMS, [M]^+^ appeared at *m/z* 341.1257 for C_19_H_19_O_5_N. Therefore, the structure of **9** was assigned as 4-hydroxyzanthacridone.

Compound **10** showed almost similar spectral data as **9** with the following exceptions: In its ^1^H NMR spectrum, the CHOH appeared downfield at δ 4.85, as compared to **9** at δ 3.63; the -CH(OH)- also appeared more downfield at δ 92.3 in **10** than at δ 78.6 in compound **9** in its ^13^C NMR spectrum. Moreover, a downfield shift was observed for C-2 from 117.5 in **9** to 160.2 in **10** in the ^13^C NMR spectrum. The relatively strong upfield shift of C-4 at δ 131.2 for oxygenated carbon with N-10 methylated acridones, was also in accordance with earlier studies [5]. Acetylation of **10** gave a downfield shift of CHOH from δ 4.85 to δ 5.35 with an additional singlet for OAc at δ 2.11 in ^1^H NMR spectrum. In the NOESY spectrum, one of the C-2’-Me at δ 1.32 correlated with C-4OH at δ 9.10 evidencing that there is no change in the position of the additional ring and happens to be quite similar as in case of **8** and **9**. Since H-4’ did not correlate with C-1 in its HMBC spectrum (as was in cases of **8** and **9**), it was evident that there is an extra bond formation by an oxide between C-2 and C-4’. This observation fits well with the value of C-2 at δ 160.2 in its ^13^C NMR spectrum. The C-4’-OH had a β-orientation as in its NOESY spectrum H-4’ showed correlation with C-1-OH (Experimental) similar to **8** and **9**. In its HRMS, it showed [M]^+^ at *m/z* 357.1210 for C_19_H_19_O_6_N. These data clearly supported the structure of **10** as 4-hydroxyzanthacridone oxide (2,4’).

### Biological evaluation

### *In vitro* antibacterial evaluation

The results of the antibacterial activity of the isolated major acridones **(1, 3, 4, 7, 8** and **10**) against three Gram-positive and one Gram-negative bacteria are given in Table 3. Compound **3** exhibited moderate activity (MIC 125 μg/mL) when tested against Gram-positive *Micrococcus luteus* and Gram-negative *Pseudomonas aeruginosa* whereas **1**, **8** and **10** showed activity against *P. aeruginosa* with MIC values of 500, 250 and 250 μg/mL, respectively. Compound **7** had MIC values of 500 and 250 μg/mL against *Staphylococcus aureus* and *Bacillus subtilis,* respectively (Table 3) while **4** displayed a MIC of 250 μg/mL against both bacteria. Against *M. luteus*, compounds **4** and **7** demonstrated a moderate activity with MIC 125 and 500 μg/mL, respectively but failed to show appreciable activity against *P. aeruginosa*. The present study related the antimicrobial activity with acridone alkaloids from the genus *Zanthoxylum,* although the acridones from the genus *Boronea* have exhibited this activity, earlier [36].

**Table 3:** ***In vitro *antibacterial activity of extracts of *Z. leprieurii*, *Z. zanthoxyloides *and acridone alkaloids**

	**MIC expressed in μg/mL**			
**Compounds**	**BS**	**ML**	**PA**	**SA**
**1**	>500	>500	500	>500
**3**	nt	125	125	nt
**4**	250	125	>500	250
**7**	250	500	>500	500
**8**	nt	>500	250	nt
**10**	nt	>500	250	nt
Kanamycin	0.53	4.16	62.5	6.94
Extracts				
*Z. leprieurii*	>1000	250	1000	>1000
*Z. zanthoxyloides*	>1000	500	>1000	>1000

Gram-positive bacteria: BS = *Bacillus subtilis* MTCC121, ML *= Micrococcus luteus* MTCC2470 and SA = *Staphylococcus aureus* MTCC96, Gram-negative bacteria: PA = *Pseudomonas aeruginosa* MTCC741, nt = not tested. Kanamycin is the standard antibiotic.

### Pharmacophore modeling and molecular docking studies against Glycosyltransferase (WAAG)

**Figure 2:**
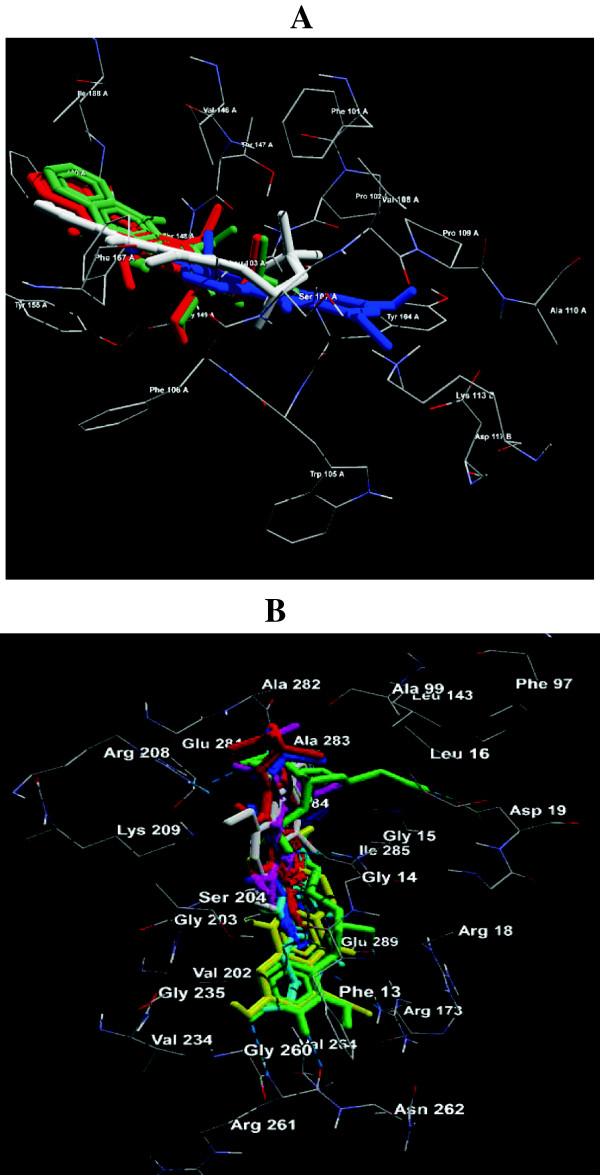
**Molecular docking of active acridone alkaloids against (A) anticancer target protein aromatase in human (compounds 1, 8, 9 and 10) and (B) antibacterial WAAG protein of bacteria (compounds 1, 2, 3, 4, 9 and 10).**

Since compounds **3** and **4** showed a MIC of 125 μg/mL against *M. luteus*, we carried out a quantitative SAR and molecular docking to explore the possible mechanism of action for the observed biological activities targeting glycosyltransferase. Results of pharmacophore modeling showed that compounds **1, 2, 3, 4, 9** and **10** have significant activity against *M. luteus*. Molecular docking study of these compounds against WAAG protein, a glycosyltransferase involved in lipopolysaccharide biosynthesis (PDB ID: 2IW1) [37], revealed active conformation and interacting binding site amino acid residues (Figure 2). For validation of molecular docking co-crystallised uridine derivative was re-docked on bacterial protein WAAG (PDB: 2IW1) (Table 4). Compounds **3** and **4** were experimentally validated to be significantly active against *M. luteus*. Quantitative SAR studies showed that compounds **2** and **9** seem to be significantly active against *M. luteus*. However, experimentally non-active compounds **7** and **8** also predicted inactive against *M. luteus*. Moreover, compounds **1** and **10** were also predicted to be inactive against *M. luteus* (Table 5).

**Table 4:** Results of molecular docking experiments targeting antibacterial protein WAAG

**Molecule**	**H-bonds**	**Bound amino acid residues of WAAG**	**Binding affinity (kcal/mol)**
	16	ASP-19, ILE-285, GLY-284, LYS-209, ALA-283, GLU-281, ARG-261, GLY-15, ARG-208, ARG-18, ARG-173, GLU-289	-10.7
Uridine-5’-diphosphate-2-deoxy-2-fluoro-alpha- d-glucose (Control inhibitor)			
	03	ARG-173, LYS-209, ARG-261	-8.6
Compound 1			
	02	ARG-173, GLY-289	-8.5
Compound 2			
	06	PHE-13, ALA-99, SER-204, GLY-284, ILE-285,	-7.6
Compound 3			
	01	PHE-13	-7.0
Compound 4			
	07	ARG-208, VAL-286, GLY-281, ALA-283, GLY-284, ILE-285,	-8.6
Compound 9			
	03	GLY-15, GLY-281, ILE-285	-8.1
Compound 10			

**Table 5:** **Derived pharmacophore model for antibacterial activity of acridones against *M. luteus***

**Acridones**	**Score obtained**	**Categorizing through threshold (S = 4.1466)**	**Predicted activity**	**Experimental activity (MIC in μg/mL)**	**Experimental response**
**1.**	4.668387	>4.1466	significantly active	>500	Non active
**2.**	4.655514	>4.1466	significantly active	Not tested	Not tested
**3.**	4.409138	>4.1466	significantly active	125	Active
**4.**	5.006913	>4.1466	significantly active	125	Active
**5.**	3.892896	<4.1466	Non-significant activity	Not tested	Not tested
**6.**	4.049106	<4.1466	Non-significant activity	Not tested	Not tested
**7.**	2.944231	<4.1466	Non-significant activity	500	Less active
**8.**	4.008224	<4.1466	Non-significant activity	>500	Non active
**9.**	4.189883	>4.1466	significantly active	Not tested	Not tested
**10.**	4.479721	>4.1466	significantly active	>500	Not active

The threshold score (S) of 4.1466 has been selected for the screening of lead compounds.

### *In vitro* cytotoxicity evaluation

We screened compounds **1**, **4, 7** and **8** for their *in vitro* cytotoxicity (Table 6) against liver (WRL-68), colon (CaCO2), breast (MCF-7) and prostate (PC-3) cancer cell lines using the MTT assay. We found that compounds **4** and **7** failed to produce a significant activity against the tested human cancer cell lines. However, a previous study [5] has reported **7** having a moderate antiproliferative activity against lung carcinoma and colorectal adenocarcinoma cell lines indicating that **7** possesses a selective cytotoxic activity. Compound **1** showed a moderate activity (IC_50_ of 86 μM) against WRL-68; both **1** and **8** exhibited a poor activity against MCF-7 with IC_50_ 229 and 205 μM, respectively.

**Table 6:** ***In vitro *cytotoxicity of acridone 1, 4, 7 and 8**

	**IC**_**50 **_**expressed in μM**			
**Compounds**	**WRL-68**	**CaCO2**	**MCF-7**	**PC-3**
**1**	86	>300	229	272
**4**	>300	>300	>300	>300
**7**	>300	>300	>300	>300
**8**	>300	>300	205	>300
Doxorubicin	1.4	6.0	3.6	8.6

WRL-68 *= Liver cancer cell lines*, CaCO2 = *colon cancer*, MCF-7 = *breast cancer,* PC-3 = *prostate cancer*. Doxorubicin Sigma D-1515 is the standard used.

### Pharmacophore modeling and molecular docking studies against aromatase and quinone Reductase

Results of pharmacophore modeling showed that compounds **1, 8, 9** and **10** have significant activity against human anticancer target aromatase [38]. Molecular docking on human aromatase, quinone reductase, not on helicase [39], was preferred because it is the popular enzyme in vertebrates known to catalyse the biosynthesis of all oestrogens from androgens and is therefore considered as a frontline therapy for oestrogen-dependent breast cancer [38]. Molecular docking study of studied compounds (**1, 8, 9, 10**) against anticancer targets aromatase (PDB: 3EQM), quinone reductase (PDB: 3G5M) showed active conformations comparable to control molecule. During docking experiments interacting binding site amino acids were namely, LEU-103, TYR-104, SER-107, GLY-149 for compound **1** with docking energy -6.1 kcal/mol and four H-bonds. Similarly, THR-148, TYR-155, SER-164 residues were identified for compound **8** with docking energy -6.7 kcal/mol and four H-bonds. Likewise residues PHE-106, THR-147, THR-148, TYR-155, SER-164 were identified in the binding site for compound **9** with docking energy -7.3 kcal/mol and showed six H-bonds. Compound **10** showed interaction with LEU-103, PHE-106, THR-147 with docking energy -7.7 kcal/mol and three H-bonds (Table 7, Figure 2). All compounds (**1, 8, 9, 10**) showed strong binding affinity with aromatase when compared with control molecule. Control experiment showed binding site residues namely, PHE-17, THR-147 with docking energy -5.8 kcal/mol and two H-bonds. For validation of docking experiment, co-crystallised inhibitor 6-methoxy-9-methyl[1,3]dioxolo[4,5-h]quinolin-8(9 h)-one was used as control molecule targeting anticancer protein aromatase (PDB: 3EQM), quinone reductase (PDB: 3G5M) (Table 7). Compounds **1** and **8** were experimentally validated to be significantly active against MCF-7 human breast cancer cell line. Results showed that out of ten compounds four showed significant anticancer activity against MCF-7 (Table 8). Besides, all studied compounds showed compliance with Lipinski's rule of five parameters for drug likeness and oral bioavailability (Table 9).

**Table 7:** Results of molecular docking experiments targeting anticancer protein uinine reductase (PDB: 3G5M) and aromatase (PDB: 3EQM)

**Molecule**	**H-bonds**	**Bound amino acid residues**	**Binding affinity (kcal/mol)**
	2	ASP-309, MET-374	-9.5
Androstenedione (control inhibitor)			
	02	PHE-17, THR-147	-5.8
6-methoxy-9-methyl[1,3]dioxolo[4,5-h]quinolin- 8(9 h)-one (Control inhibitor)			
	04	LEU-103, TYR-104, SER-107, GLY-149	-6.1
Compound 1			
	04	THR-148, TYR-155, SER-164	-6.7
Compound 8			
	06	PHE-106, THR-147, THR-148, TYR-155, SER-164	-7.3
Compound 9			
	03	LEU-103, PHE-106, THR-147	-7.7
Compound 10			

**Table 8:** Derived pharmacophore model for anticancer activity of acridones active against human cancer cell line MCF-7

**Acridones**	**Score obtained**	**Categorizing through threshold (S = 4.1274)**	**Predicted activity**	**Experimental activity (IC**_**50 **_**in μM)**	**Experimental response**
1.	4.2712	> 4.1274	significantly active	229	Poor activity
2.	3.5826	< 4.1274	Non-significant activity	Not tested	Not tested
3.	3.6316	< 4.1274	Non-significant activity	Not tested	Not tested
4.	3.6796	< 4.1274	Non-significant activity	>300	Non active
5.	3.3015	< 4.1274	Non-significant activity	>300	Non active
6.	2.9402	< 4.1274	Non-significant activity	Not tested	Not tested
7.	2.4587	< 4.1274	Non-significant activity	Not tested	Not tested
8.	5.1457	> 4.1274	Significantly active	205	Poor activity
9.	4.9482	> 4.1274	Significantly active	Not tested	Not tested
10.	5.5092	> 4.1274	Significantly active	Not tested	Not tested

The threshold score (S) of 4.1274 has been selected for the screening of lead compounds.

**Table 9:** Compliance of acridone alkaloids with standard parameters of Lipinski’s rule of five for drug likeness and oral bioavailability

**Acridone alkaloids**	**miLOGP**	**MW**	**nON**	**nOHNH**	**nviolations**
**1**	1.926	301.298	6	2	0
**2**	1.862	257.245	5	3	0
**3**	1.119	303.27	7	4	0
**4**	2.189	285.299	5	1	0
**5**	2.433	255.273	4	1	0
**6**	1.913	271.272	5	2	0
**7**	2.173	255.273	4	1	0
**8**	2.476	355.39	6	2	0
**9**	2.2	341.363	6	3	0
**10**	2.411	357.362	7	3	0

**miLogP** = octanol/water partition coefficient, **MW** = molecular weight, **nON** = number of hydrogen acceptor, **nOHNH =** number of hydrogen donor, **nviolations** = violations from Lipinski’s rule.

## Experimental

### General experimental procedures

^1^H and ^13^C NMR spectra were obtained with a FT-NMR 300 MHz equipped with a 5 mm ^1^H and ^13^C (ATP) probe operating at 300 and 75 MHz, respectively, with TMS as internal standard. Chemical shifts were reported in δ (ppm) and coupling constants (J) were measured in Hz. QTOF-HRMS spectra were recorded on Agilent 6520-QTOF LC/MS having an ESI source in positive mode. Dry Nitrogen was used with 11 LPM flow rate for ionization at 350 Dc. The ESIMS was recorded on API-3000 LC-MS/MS ABSCIEX in 1.0 ppm solution through MS grade acetonitrile-water with injection mode: infusion through motor driven hardware syringe. UV analysis was carried out on a Spectronic Genesys 2 spectrophotometer and IR was recorded with FT-IR Perkin Elmer instrument. Optical rotations were taken with a Horiba SEPA-300 polarimeter. Melting points were determined on a Fisher-Johns melting point apparatus and were uncorrected. Precoated aluminium sheets silica gel 60 F254 TLC plates were used to check the purity of compounds and preparative TLC was performed on glass plates of silica gel 60 (20×20 cm, Merck). Flash chromatography was performed with a Buchi Pump manager C-615 flash model operating with two pump modules C-605. Spots were viewed under UV lamp (254 nm and 365 nm) or sprayed with Anisaldehyde- sulfuric acid and Draggendorff reagents. All reagents used, were of analytical grades.

### Plant material

The dried fruits of *Z. leprieurii* and *Z. zanthoxyloides* were collected at Douala, Littoral Province of Cameroon in December 2009. The plants were identified at the University of Douala (TF) and the samples were compared with the voucher specimen number 2713/SRFK/CAM and 21793/SFR/CAM for *Z. leprieurii* and *Z. zanthoxyloides*, respectively at the National Herbarium of Cameroon, Yaounde.

### Extraction and isolation

#### Extraction and isolation from *Z. zanthoxyloides*

The dried and ground fruits (400 g) were successively extracted under percolation at room temperature overnight with *n*-hexane three times. Thereafter, they were extracted by methanol at room temperature overnight which gave 16 g of crude extracts. A portion of this extract (10 g) was dissolved in CHCl_3_ (30 mL) and extracted three times with 5 N HCL. The aqueous layer was basified with 25% ammonium hydroxide and the basic alkaloidal part was extracted with CHCl_3_ (3×100 mL). The extract was dried over anhydrous sodium sulfate and solvent was evaporated. The extract (240 mg) obtained as above, after silica gel column chromatography (58 g, 60-120 mesh, 2.3 × 93 cm) with the mixtures of *n*-hexane, EtOAc and MeOH in the increasing order of polarity in basic medium (2 drops ammonium hydroxide/100 mL solvent), afforded 16 fractions. From fraction 8, after preparative TLC, **4 (**5 mg) was obtained. While fraction 9 (*n*-hexane-EtOAc, 1:4) was left to crystallize at room temperature to obtain orange crystals of **3** (3.5 mg, TLC, EtOAc- CHCl_3_- MeOH, 97:1:2, Rf 0.24), fraction 10 eluted with *n*-hexane-EtOAc (1:4 and 1:9) was further purified through preparative TLC to afford **2** (6 mg, TLC, MeOH + 1% NH_4_OH- C_6_H_6_- CH_2_Cl_2,_ 1:2:2, Rf 0.64). Fractions (11-13) which were complex mixtures were pooled together and purified through flash chromatography (20 g, 230-400 mesh, 15 × 100 mm glass column C-690 Sepacore Buchi fitted with a precolumn of ID 15 mm) with EtOAc as solvent A and MeOH plus 2 drops ammonium hydroxide/100 mL as solvent B. The flow rate was set up to 2.5 mL/min. A total of 52 fractions (each of 12 mL) were collected and pooled according to their TLC pattern into four fractions. Fractions 1 and 2 gave yellowish crystals of **1 (**32 mg, TLC, EtOAc- MeOH + 1% NH_4_OH, 9:1, Rf 0.71), **8** (19.2 mg, TLC, EtOAc- MeOH+ 1% NH_4_OH, 9:1, Rf 0.67) and **5**. Fraction 3 afforded **9** (10.3 mg, TLC, EtOAc- MeOH + 1% NH_4_OH, 9:1, Rf 0.53) and fraction 4 yielded **10 (**22 mg, TLC, EtOAc- MeOH + 1% NH_4_OH 9:1, Rf 0.49). All the isolated alkaloids gave yellowish orange colored crystals or crystalline yellow mass and positive test with Dragendorff reagent.

### Extraction and isolation from *Z. leprieurii*

The dried fruits (380 g) after extraction as described above, yielded 48 g of methanol extract from which 40 g was dissolved in CHCl_3_ (110 mL) and the alkaloids extracted as detailed above. The silica gel cc (120 g, 60-120 mesh, 2.5 × 100 cm) yielded complex mixtures (340 mg) which after flash chromatography (116 g, 230-400 mesh, 36 × 230 mm glass column C-690 Sepacore Buchi) using *n*-Hexane as solvent A and EtOAc plus 2 drops ammonium hydroxide/100 mL as solvent B with the flow rate stated as above, afforded eight fractions. Recrystallisation of fractions 3, 5 and 6 in MeOH- CHCl_3_ afforded **7** (10 mg), **1** (3 mg) and **4** (40 mg), respectively. Compound **6** (5 mg) was obtained from fraction 7, after recrystallization in MeOH- CHCl_3_.

### Spectroscopic data of new compounds

**3-Hydroxy-1,5,6-trimethoxy-9-acridone** (**1**): Mp: 165°C; UV (CHCl_3_) λmax (log ϵ) nm: 233 (0.781), 252 (2.511), 320 (0.426); IR (KBr) ν_max_ cm^-1^: 3448, 2929, 1620, 1579, 1507; ^1^H and ^13^C NMR see Tables 1 and 2; ^1^H^1^H COSY: H-8 at δ 7.99 **―** H-7 at δ 7.21; HMBC: C-3-OH → C-3, C-2, C-4; C-5-OMe → C-5; C-6-OMe → C-6; ESIMS *m/z* (rel. int.): 301 (5) [M]^+^, 282 (100), 260 (82), 227 (30), 202 (42); HRMS: 301.0944 (Calc. for C_16_H_15_O_5_N 301.0945).

**1,6-Dihydroxy-3-methoxy-9-acridone** (**2**): UV (CHCl_3_) λmax (log ϵ) nm: 251 (2.011), 321 (0.406); IR (KBr) ν_max_ cm^-1^: 3438, 2929, 1620, 1579; ^1^H and ^13^C NMR see Tables 1 and 2; ^1^H^1^H COSY: H-8 at δ 7.89 **―** H-7 at δ 7.07; HMBC: C-3-OMe → C-3; ESIMS *m/z* (rel. int.): 257 (15) [M]^+^, 245 (60), 214 (100); HRMS: 257.0681 (Calc. for C_14_H_11_O_4_N 257.0683).

**3,4,5,7-Tetrahydroxy-1-methoxy-10-methyl-9-acridone** (**3**): Mp > 250°C; UV (CHCl_3_) λmax (log ϵ) nm: 262 (2.511), 320 (0.426); IR (KBr) ν_max_ cm^-1^: 3430, 2929, 1620, 1580; ^1^H and ^13^C NMR see Tables 1 and 2; HMBC: C-1-OMe → C-1; H-2 → C-2, C-1, C-3; ESIMS *m/z* (rel. int.): 303 (5) [M^+^], 298 (8), 286 (22), 282 (100); HRMS: 303.0734 (Calc. for C_15_H_13_O_6_N 303.0737).

**4-Methoxyzanthacridone** (**8**): [α]_D_^22^: -3.69^o^ (CHCl_3_, c = 0.22); UV (CHCl_3_) λmax (log ϵ) nm: 243 (1.898), 275 (1.204), 327 (1.005); IR (KBr) ν_max_ cm^-1^: 3368, 2929, 1654, 1620, 1579; ^1^H and ^13^C NMR see Tables 1 and 2; ^1^H^1^H COSY: H-8 at δ 7.82 **―** H-7 at δ 7.29 **―** H-6 at δ 7.61 ― H-5 at δ 7.40; H-4’ at δ 3.50 ― H-3a’ at δ 2.72 ― H-3b’ at δ 3.07; HMBC: H-4’ à C-2, C-1, C-2’, C-3’; H-3a’ and H-3b’à C-2, C-4’, C-2’; C-2’Me àC-2’, C-3’; NOESY: H4’― C-1-OH, H-3’a, H-3’b; ESIMS m/z (rel. int.): 355 (2) [M]+, 330 (18), 314 (100), 202 (8); HRMS: 355.1416 (Calc. for C20H21O5N 355.1414).

### Acetylation of 4-methoxyzanthacridone (8)

Using 1 mL of acetic anhydride and one drop of pyridine, **8** (2 mg) was acetylated and left for reflux at ambient temperature for 6 hrs. The reaction mixture after solvent evaporation under rotavapor yielded compound **8a. **^1^H NMR (CDCl_3_, δ): 4.34 (dd, J = 10.0, 3.5 Hz) H4’, 2.10, s, OAc.

**4-Hydroxyzanthacridone** (**9**): [α]_D_^22^: -4.11^o^ (CHCl_3_, *c* = 0.11); UV (CHCl_3_) λmax (log ϵ) nm: 243 (1.826), 318 (0.618), 329 (0.562); IR (KBr) ν_max_ cm^-1^: 3360, 2931, 1652, 1620, 1581; ^1^H and ^13^C NMR see Tables 1 and 2; ^1^H^1^H COSY: H-8 at δ 8.40 **―** H-7 at δ 7.34 **―** H-6 at δ 7.59 **―** H-5 at δ 7.41; H-4’ at δ 3.63 **―** H-3a’ at δ 2.68 **―** H-3b’ **―** at δ 3.00; HMBC: H-4’ → C-2, C-1, C-2’, C-3’; H-3a’and H-3b’ → C-2, C-4’, C-2’; C-2’Me →C-2’, C-3’; NOESY: H-4’—C-1-OH, H-3’a, H-3’b; ESIMS *m/z* (rel. int.): 341 (2) [M]^+^, 296 (32), 282 (100), 260 (35), 224 (40); HRMS: 341.1257 (Calc. for C_19_H_19_O_5_N 341.1258).

### Acetylation of 4-hydroxyzanthacridone (9)

Using 1 mL of acetic anhydride and one drop of pyridine, **9** (2 mg) was acetylated and left for reflux at ambient temperature for 6 hrs. The reaction mixture after solvent evaporation under rotavapor yielded compound **9a. **^1^H NMR (CDCl_3_, δ): 4.22 (dd, J = 10.0, 3.5 Hz) H-4’, 2.10, s, OAc.

**4-Hydroxyzanthacridone oxide (2,4’)** (**10**): [α]_D_^22^: -1.32^o^ (CHCl_3_, *c* = 0.25); UV (CHCl_3_) λmax (log ϵ) nm: 242 (1.247), 312 (0.507); IR (KBr) ν_max_ cm^-1^: 3423, 1654, 1623, 1582; ^1^H and ^13^C NMR see Tables 1 and 2; ^1^H^1^H COSY: H-8 at δ 8.39 **―** H-7 at δ 7.34 **―** H-6 at δ 7.55 **―** H-5 at δ 7.34; H-4’ at δ 4.85 **―** H-3a’ at δ 3.23 and H-3b’ at δ 3.25; HMBC: H4’ → C-2, C-2’, C-3’; H-3a’and H-3b’ → C-4’, C-2’; C-2’Me → C-2’, C-3’; NOESY: H-4’**―** C-1-OH, H-3’a, H-3’b; C-2’Me— C-4OH; ESIMS *m/z* (rel. int.): 357 (2) [M]^+^, 316 (28), 296 (72), 282 (100), 260 (48); HRMS: 357.1210 (Calc. for C_19_H_19_O_6_N 357.1207).

### Acetylation of 4-hydroxyzanthacridone oxide (2,4’) (10)

Acetylation was done on 2 mg of **10** using 1 mL of acetic anhydride and one drop of pyridine and left for reflux at ambient temperature for 6 hrs. The reaction mixture after solvent evaporation under rotavapor yielded compound **10a. **^1^H NMR (CDCl_3_, δ): 5.35 (dd, J = 10.0, 3.5 Hz) H-4’, 2.11, s, OAc.

### Biological assays

#### Antibacterial assay

The four bacterial strains viz., *Micrococcus luteus* MTCC2470, *Staphylococcus aureus* MTCC96, *Bacillus subtilis* MTCC121 and *Pseudomonas aeruginosa* MTCC741 used for the assay, were acquired from Microbial Type Culture Collection (MTCC), Chandigarh, India. MIC values of the tested compounds **1**, **3**, **4**, **7, 8** and **10** against pathogens, were determined by microdilution method [40]. The inocula of microorganisms were prepared from a 17 h broth culture and the suspensions were adjusted to 0.5 Mc Farland turbidity. The tested samples were first dissolved in dimethyl sulfoxide (DMSO), then in Mueller Hinton Broth to the highest dilution of 500 μg/mL. Then, serial twofold dilutions were made in a series of concentration with 0.0078 μg/mL being the smallest concentration in the 96 wells microplate. Ampicillin and Kanamycin diluted prior in water were used as reference antibiotics. Negative control was made with DMSO.

### Cytotoxicity assay

IC_50_ values for compounds **1, 4, 7** and **8** were determined as described previously [41]. 1.5-2.0X10^3^ cells/well were incubated in the 5% CO_2_ incubator for 24 h to enable them to adhere properly to the 96-well microplates (Greiner, Germany). Test compounds dissolved in DMSO were added at varying concentrations and left for 4 h. After 48 h, 10 μL MTT (Sigma M-2128) was added and the plates were incubated for an additional 4 h. The absorbance was read on a Fluostar Omega Microplate reader (BMG LABTECH, Australia) at 570 nm within 1 h of DMSO addition and the respective IC_50_ were calculated. The experiments were done in triplicate.

### Molecular modeling and docking studies

A pharmacophore model has been derived to predict the cytotoxic and antibacterial activities of acridone alkaloids against human cancer cell line MCF-7 and *M. luteus,* respectively. Derived QSAR models have been validated using standard method [42]. For QSAR model development against MCF-7, a total of 27 known compounds were used in the training data set. These compounds showed >70% structural similarity with acridone alkaloid skeleton. For statistical relationship eight chemical descriptors namely, AlogP, BCUTw-1 l, Eccentric connectivity index, LipoaffinityIndex, McGowan volume, Petitigen number, TPSA and Zagreb index were calculated for QSAR model building. The similar approach has been used for the development of QSAR model for antibacterial activity. On the other hand, molecular docking simulation study was performed by AutoDock-Vina software [43]. Aromatase (PDB:3EQM), quinone reductase (PDB: 3G5M) and glycosyltransferase (WAAG) (PDB: 2IW1) target proteins have been used during docking experiments to reveal the binding affinity against anticancer and antibacterial targets, respectively [37,44]. Drug likeness and oral bioavailability of studied compounds were evaluated through Lipinski’s rule of five.

## Conclusions

Although acridone class of alkaloids are uncommon in nature but they have been found to be present in several Genus of Family Rutaceae. Only one specy of *Zanthoxylum, i.e. Z. leprieurii*, has recently been investigated for acridone alkaloids, in detail*.* Now, we have investigated the fruits of medicinally important species of Cameroonian *Z. zanthoxyloides* and *Z. leprieurii,* for their acridone alkaloids and confirmed their antibacterial and cytotoxic activities. The prediction of possible mechanism of action has been done through molecular modeling of the isolated compounds. Moreover, the present work of isolation of novel acridone alkaloids from *Z. zanthoxyloides* emphasizes their uniqueness as taxonomic markers among the African species of this genus. We have isolated and identified 10 acridones out of which 6 were new (**1**, **2**, **3**, **8**, **9** and **10**). Acridones **8, 9** and **10** are described with an unusual tetracyclic acridone which has now been named as zanthacridone because of their new carbon skeleton. The quantitative SAR and molecular modeling studies suggested that the compounds **1**, **9** and **10** are inhibitors of aromatase, quinone reductase and glycosyltransferase. Therefore, these compounds hold the promise for further studies to use them as chemopreventive agents and LPS inhibitors.

## Competing interests

The authors declare that they have no competing interests.

## Authors’ contributions

LM and WNAV conceived the work; WNAV collected the fruits for onward extraction and purification of compounds, the antibacterial assay and drafted the manuscript. LM identified the compounds after purification, finalized the manuscript and supervised the bench work. SK performed the *in vitro* cytotoxic activity. OP and FK did the QSAR and modeling studies. TF identified the plant material and helped in the collections. VK supervised the work and improved the manuscript. All the authors read and approved the manuscript.

## Supplementary Material

Additional file 1 **Figure 1a: **^**1**^**H NMR of compound 1. b: **^**13**^**C NMR of compound 1. c: DEPT of compound 1. d: COSY of compound 1. e: Mass of compound 1. f: IR of compound 1. Figure 2a: **^1^H NMR of compound **2. b**: Mass of compound **2. Figure 3a: **^1^H NMR of compound **3. b: **^13^C NMR of compound **3. c**: DEPT of compound **3. d:** Mass of compound **3. e**: IR of compound **3. Figure 4a**: ^1^H NMR of compound **8. b: **^13^C NMR of compound **8. c:** DEPT of compound **8. d:** COSY of compound **8. e:** NOESY of compound **8. f:** Mass of compound **8. g:** IR of compound **8. Figure 5a: **^1^H NMR of compound **9. b: **^13^C NMR of compound **9. c: **^13^C NMR of compound **9. d:** DEPT of compound **9. e:** DEPT of compound **9. f:** Mass of compound **9. Figure 6a: **^1^H NMR of compound **10. b: **^13^C NMR of compound **10. c:** DEPT of compound **10. d:** COSY of compound **10. e:** Mass of compound **10. f:** IR of compound **10.** Click here for file
