# Supplementary material for: Aromatase and glycosyl transferase inhibiting acridone alkaloids from fruits of Cameroonian Zanthoxylum species
Source: Chem Cent J. 2013 Jul 18;7:125. doi: 10.1186/1752-153X-7-125 (PMC3750308; doi:10.1186/1752-153X-7-125)
Supplement: Additional file 1 — Figure 1a: 1H NMR of compound 1. b: 13C NMR of compound 1. c: DEPT of compound 1. d: COSY of compound 1. e: Mass of compound 1. f: IR of compound 1. Figure 2a: 1H NMR of compound 2. b: Mass of compound 2. Figure 3a: 1H NMR of compound 3. b: 13C NMR of compound 3. c: DEPT of compound 3. d: Mass of compound 3. e: IR of compound 3. Figure 4a: 1H NMR of compound 8. b: 13C NMR of compound 8. c: DEPT of compound 8. d: COSY of compound 8. e: NOESY of compound 8. f: Mass of compound 8. g: IR of compound 8. Figure 5a: 1H NMR of compound 9. b: 13C NMR of compound 9. c: 13C NMR of compound 9. d: DEPT of compound 9. e: DEPT of compound 9. f: Mass of compound 9. Figure 6a: 1H NMR of compound 10. b: 13C NMR of compound 10. c: DEPT of compound 10. d: COSY of compound 10. e: Mass of compound 10. f: IR of compound 10. [file 1752-153X-7-125-S1.doc]

**
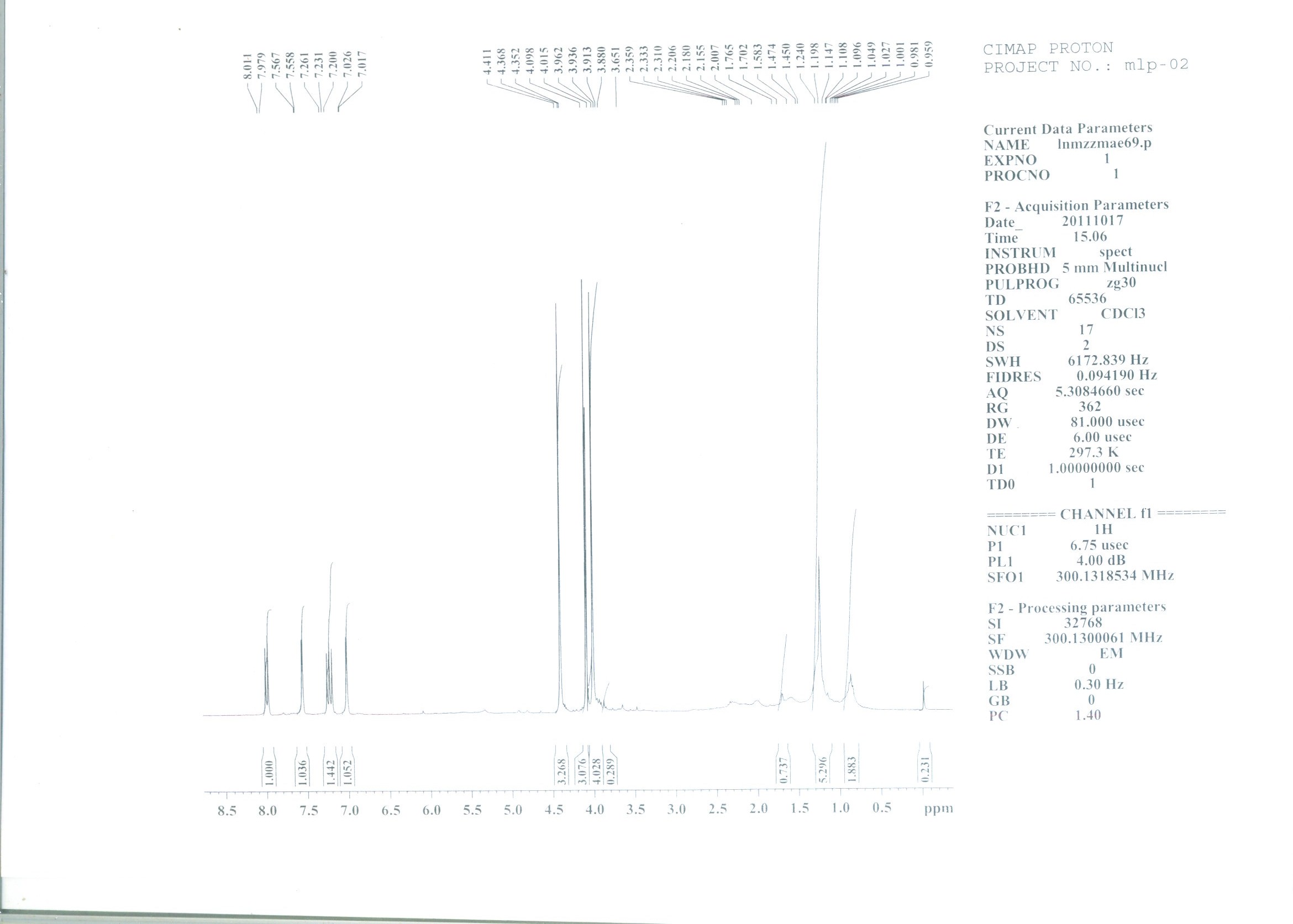
**

**Figure 1a:** ^1^H NMR of compound **1**

**
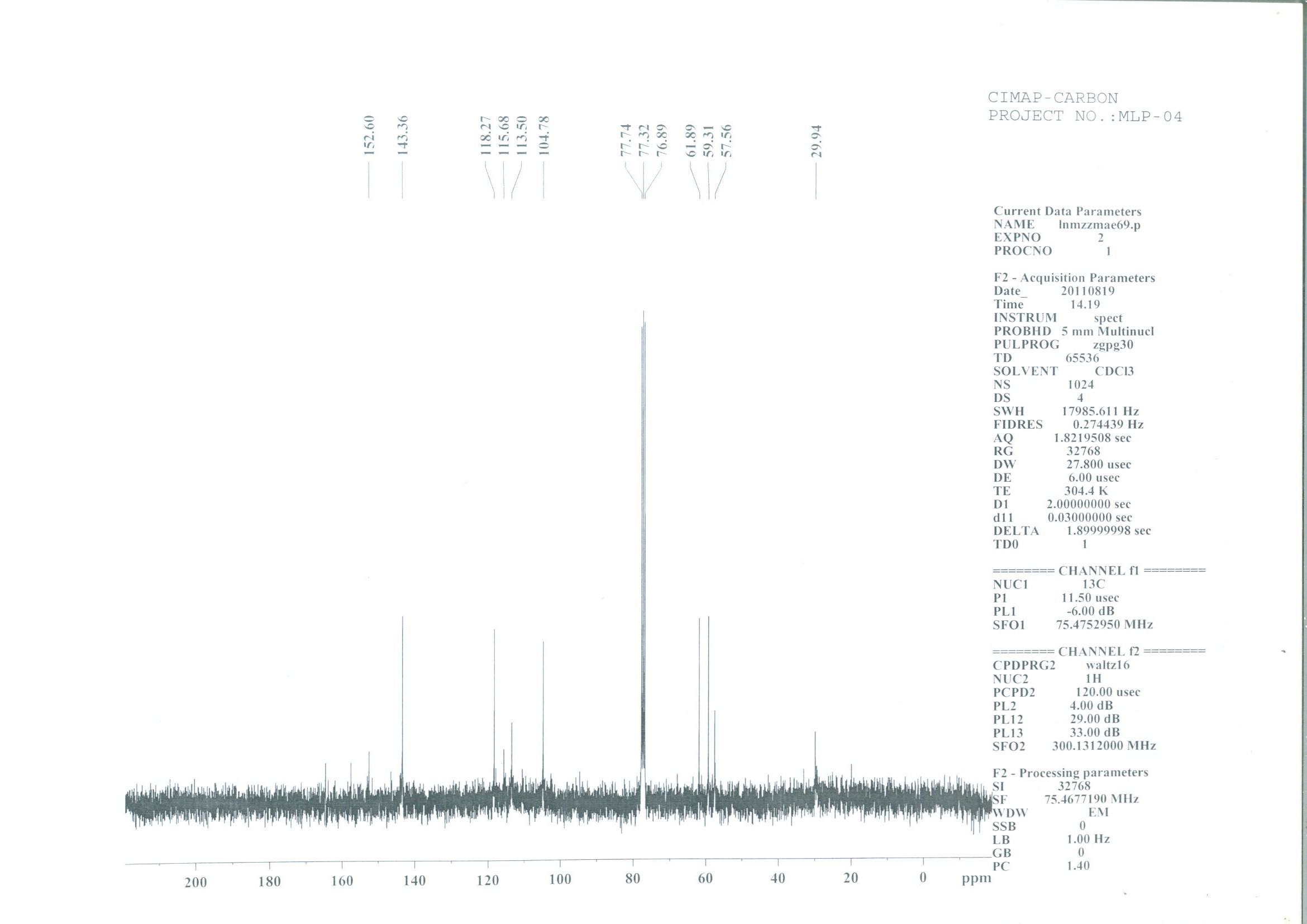
**

**Figure 1b:** ^13^C NMR of compound **1**

**
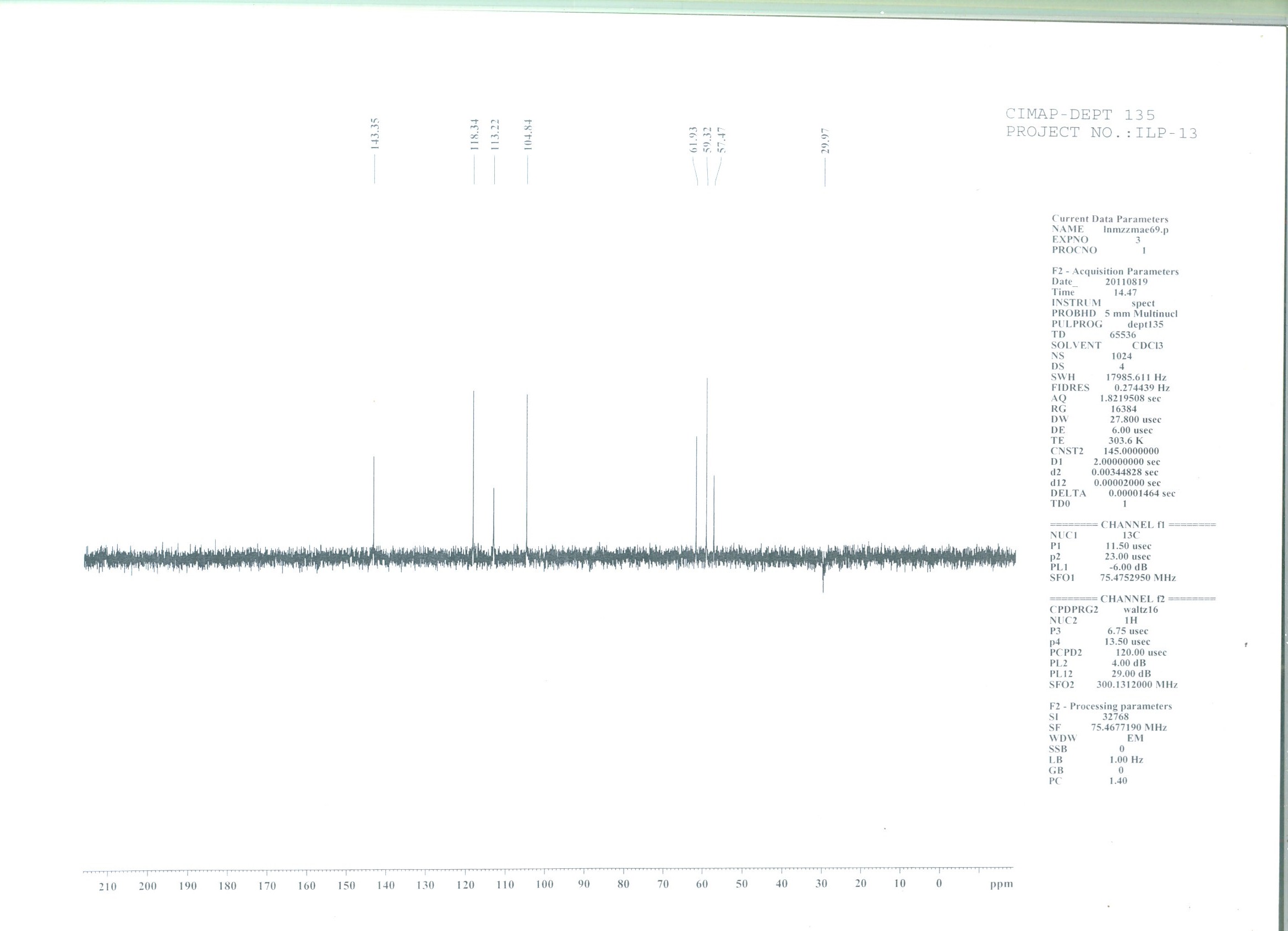
**

**Figure 1c:** DEPT of compound **1**

**
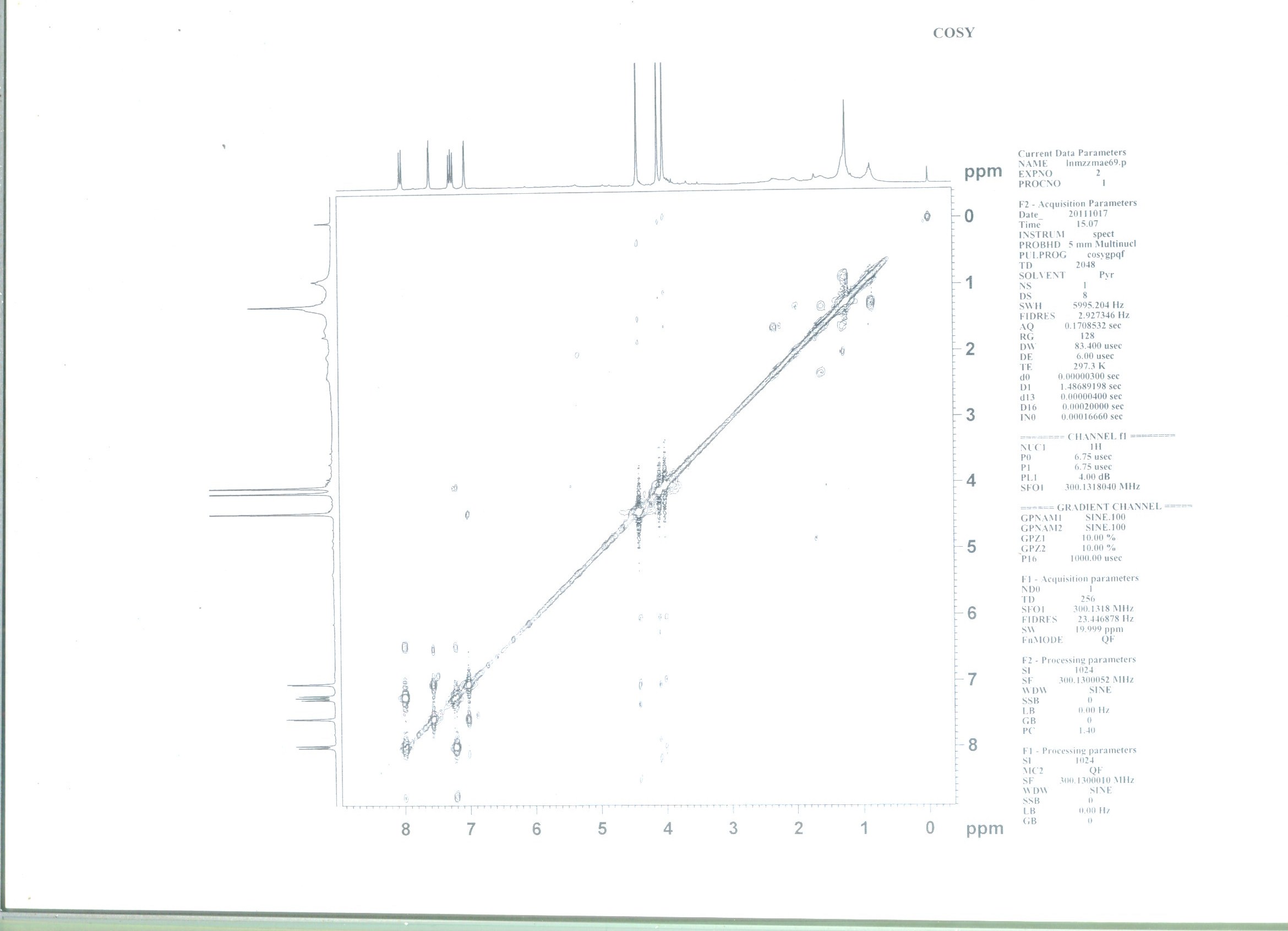
**

**Figure 1d:** COSY of compound **1**

**
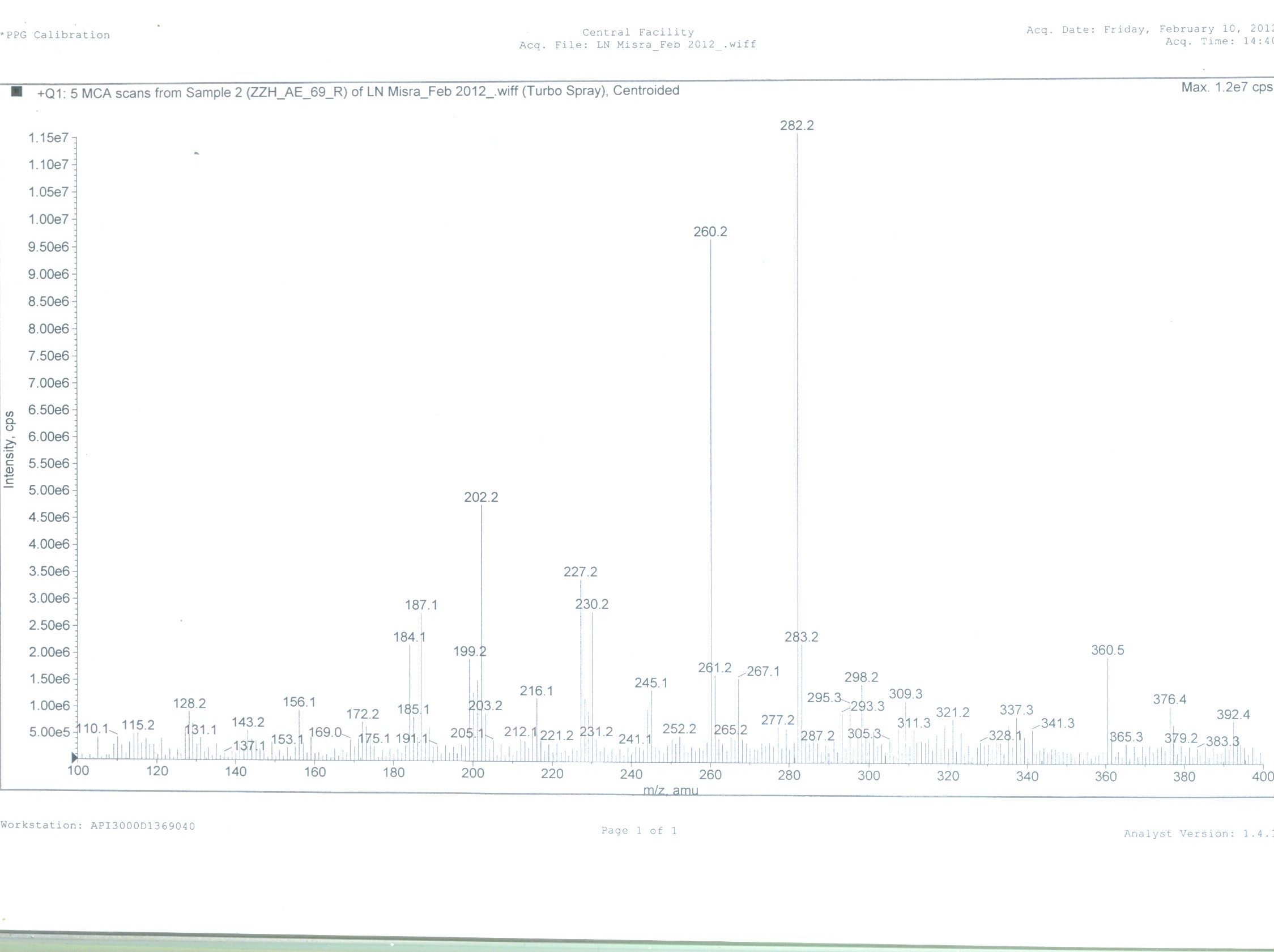
**

**Figure 1e:** Mass of compound **1**

**
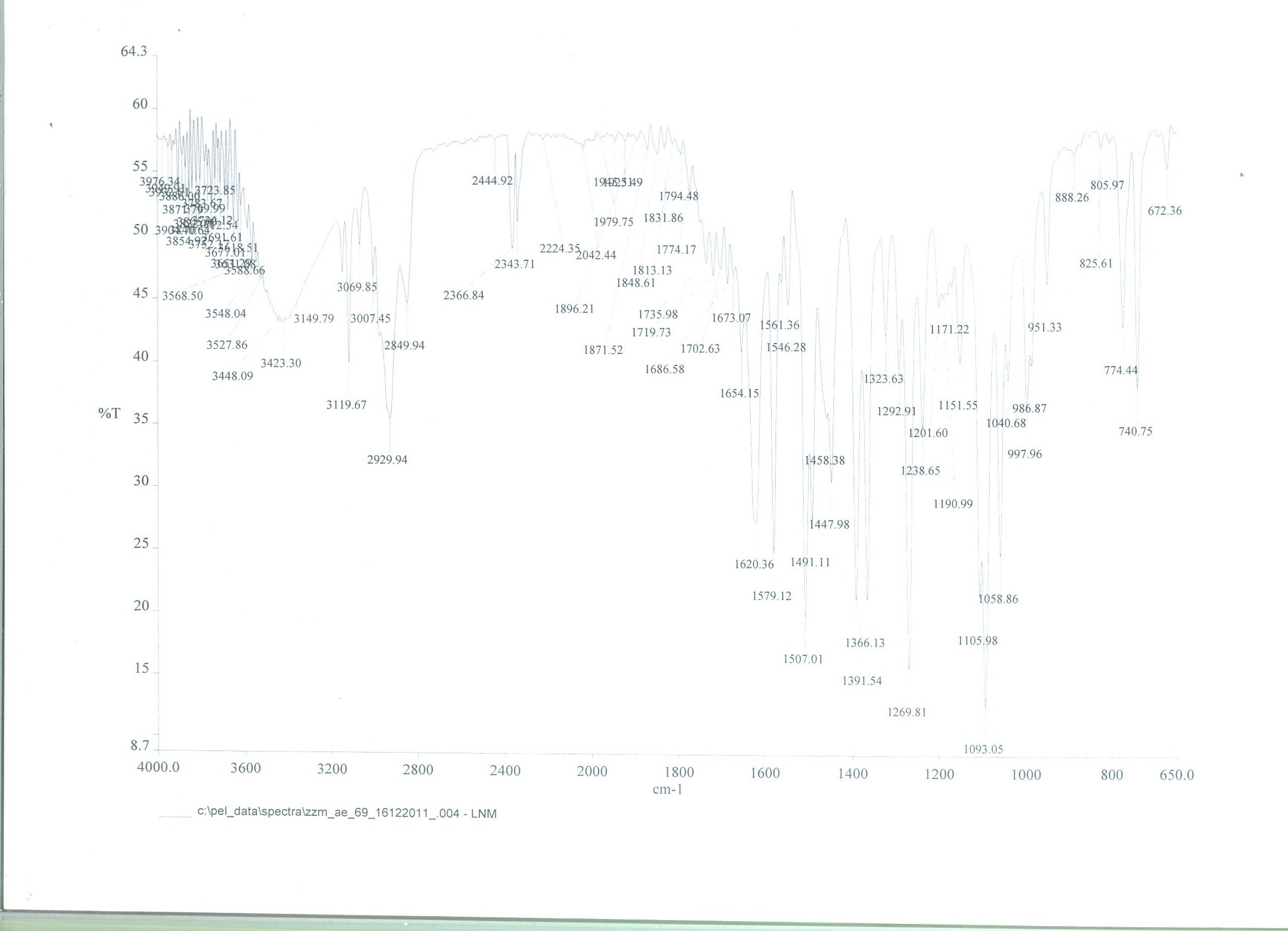
**

**Figure 1f:** IR of compound **1**

**
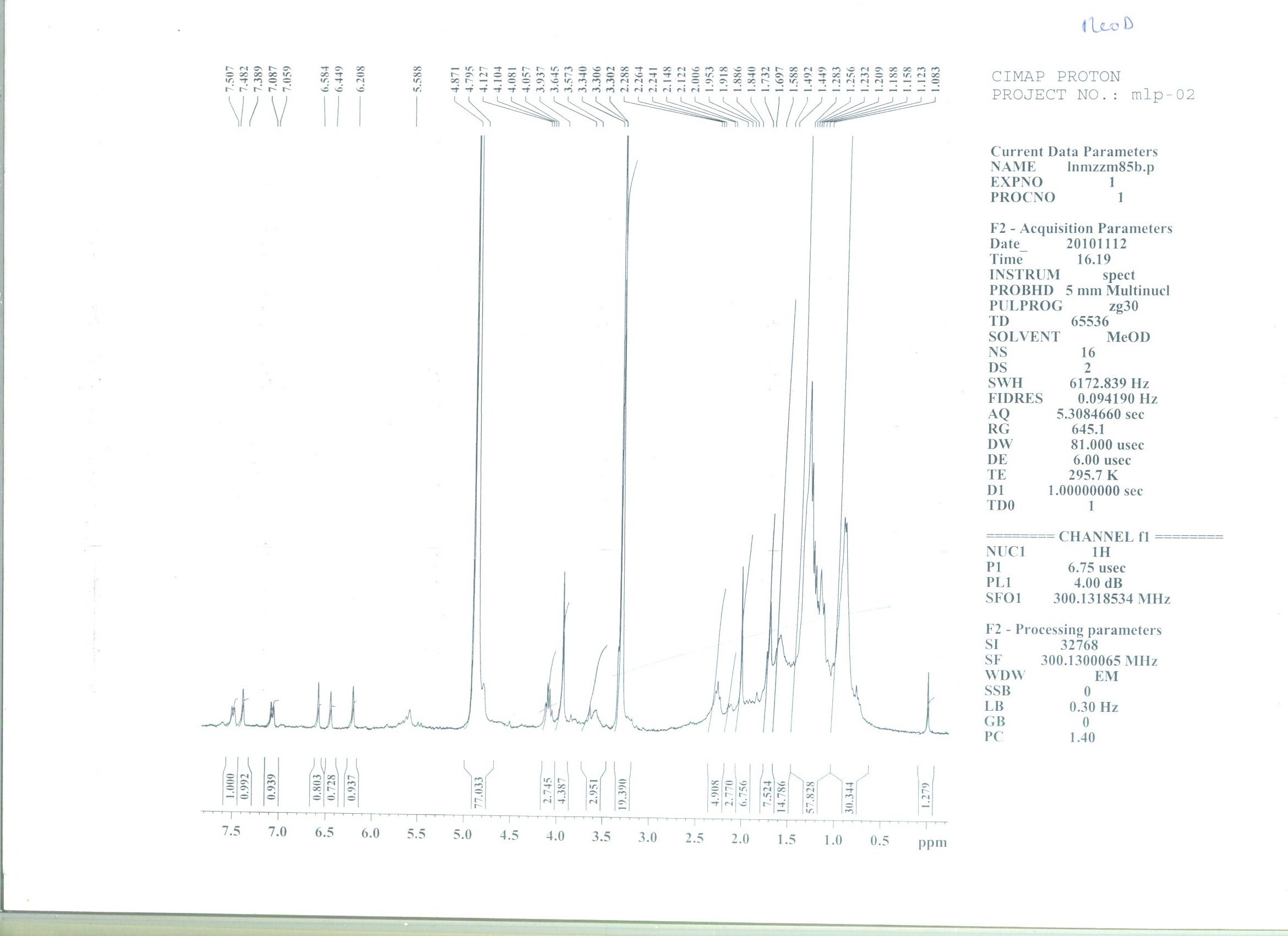
**

**Figure 2a:** ^1^H NMR of compound **2**

**
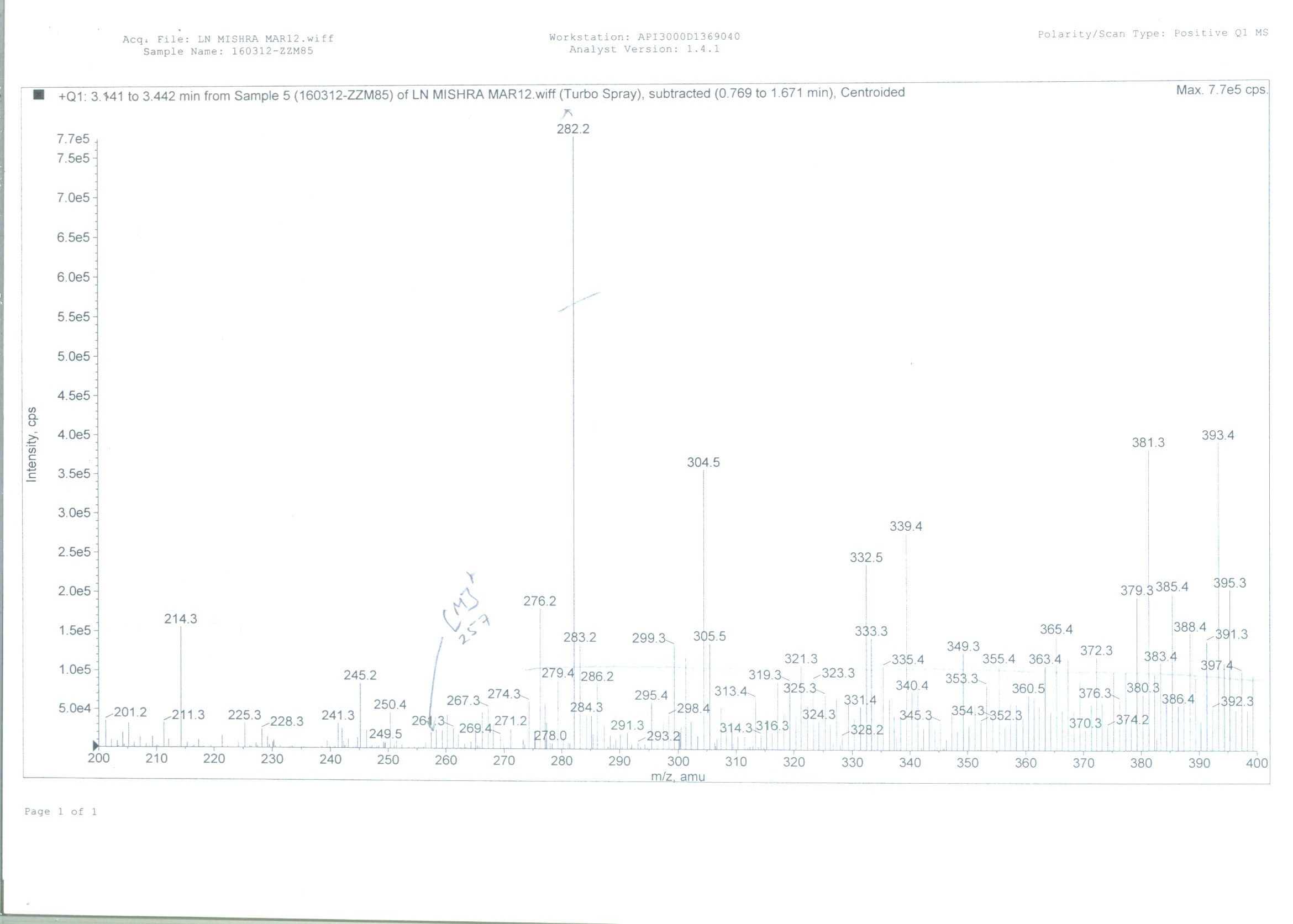
**

**Figure 2b**: Mass of compound **2**

**
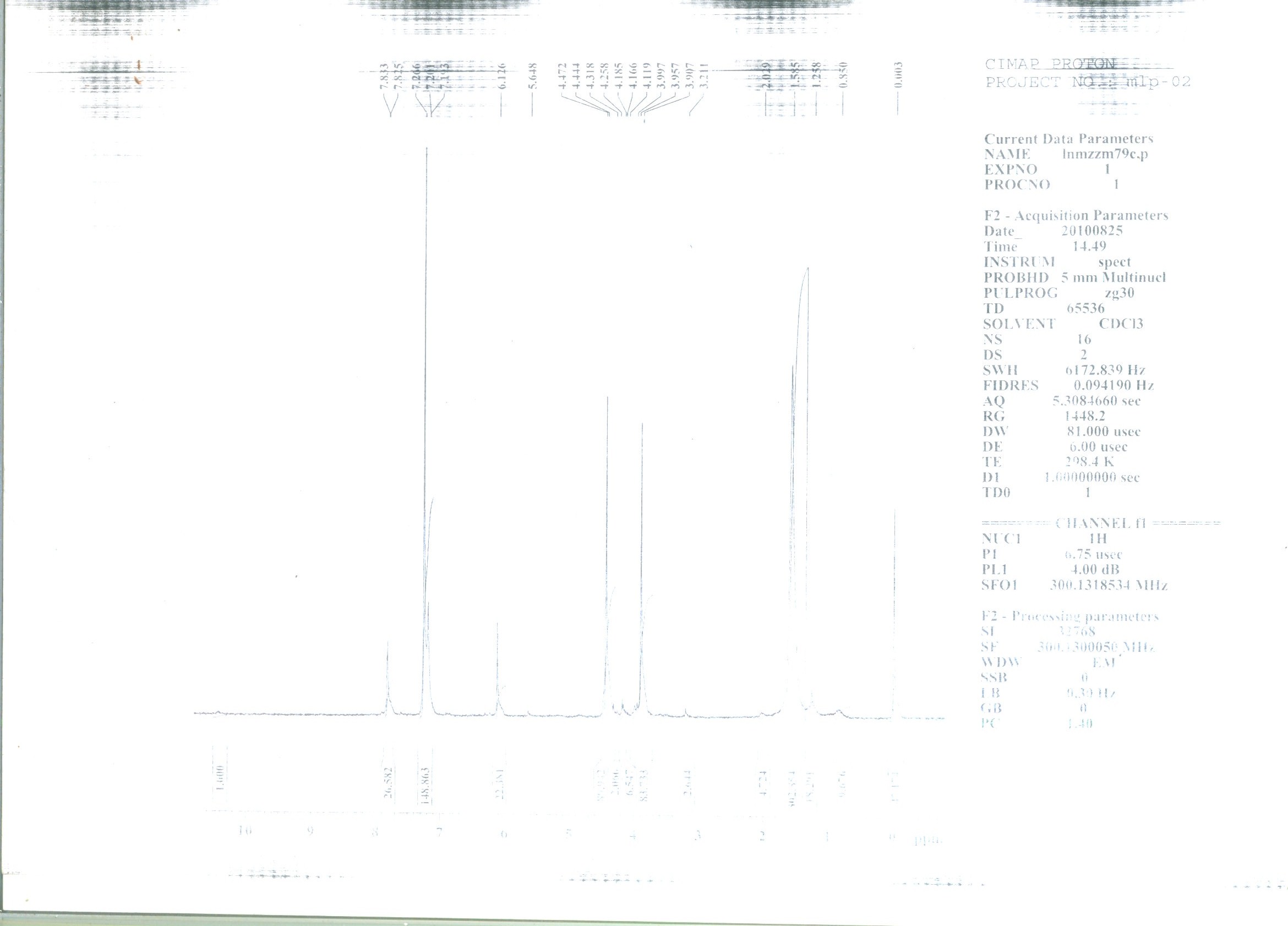
**

**Figure 3a:** ^1^H NMR of compound **3**

**
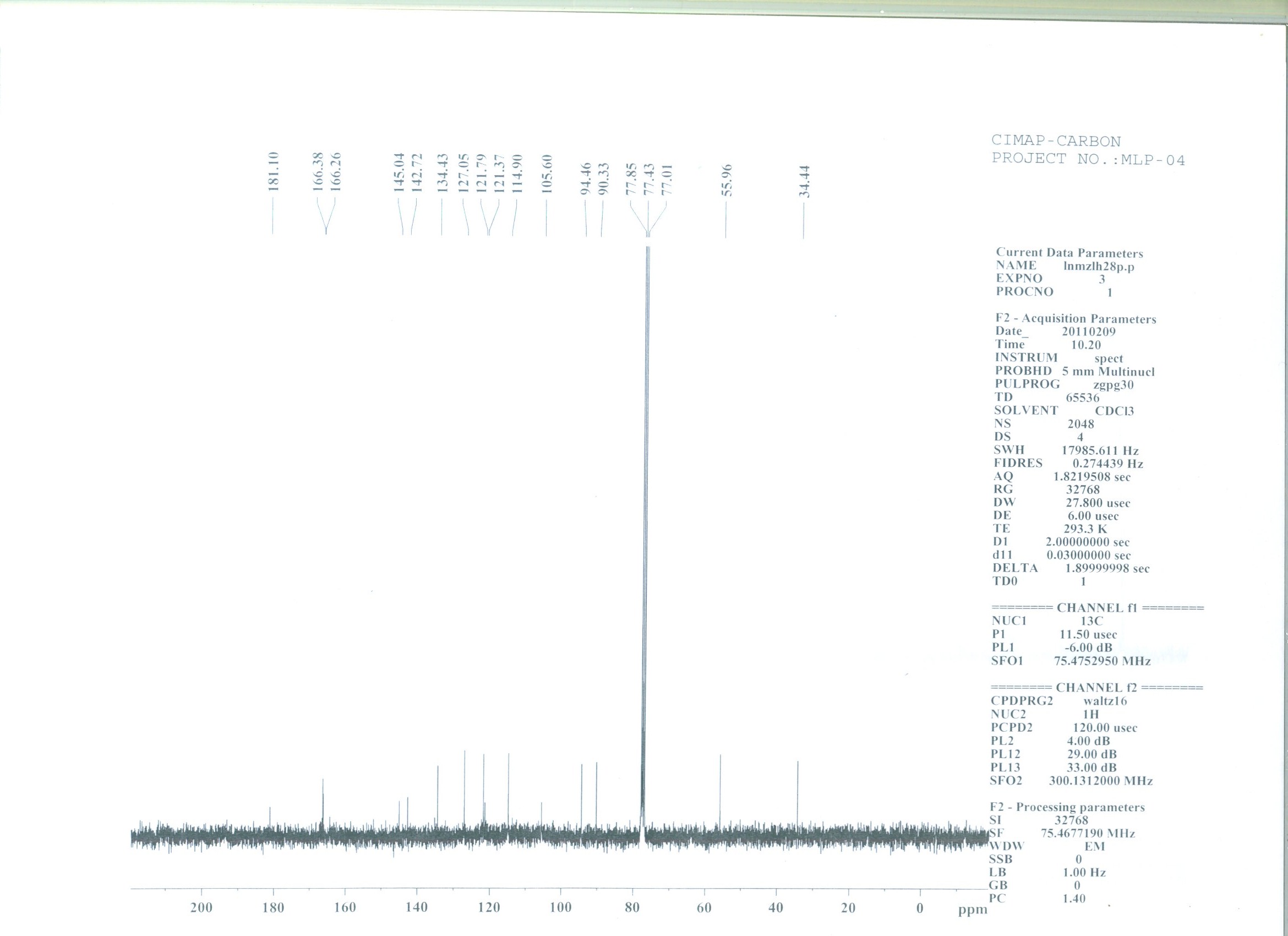
**

**Figure 3b:** ^13^C NMR of compound **3**

**
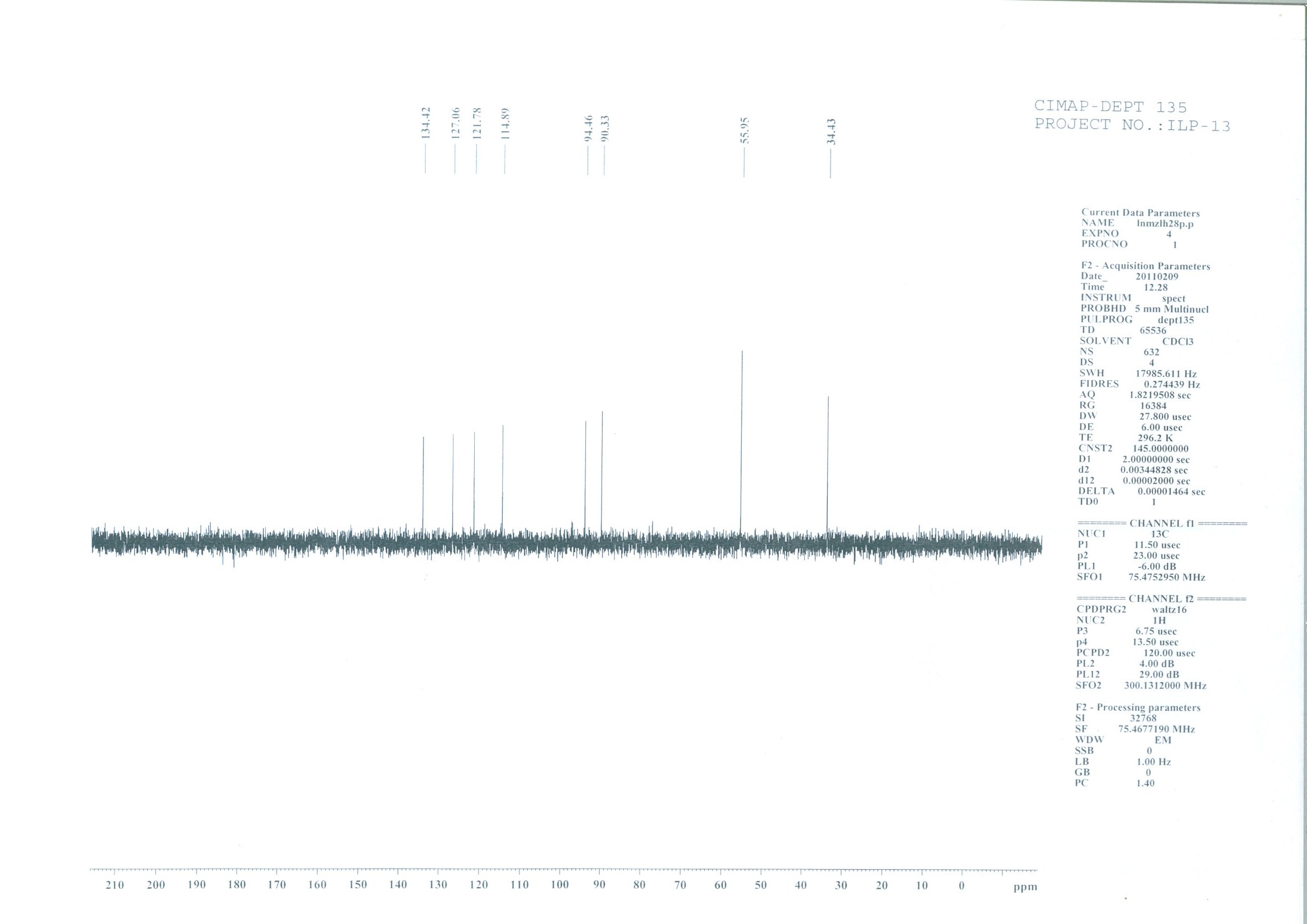
**

**Figure 3c**: DEPT of compound **3**

**
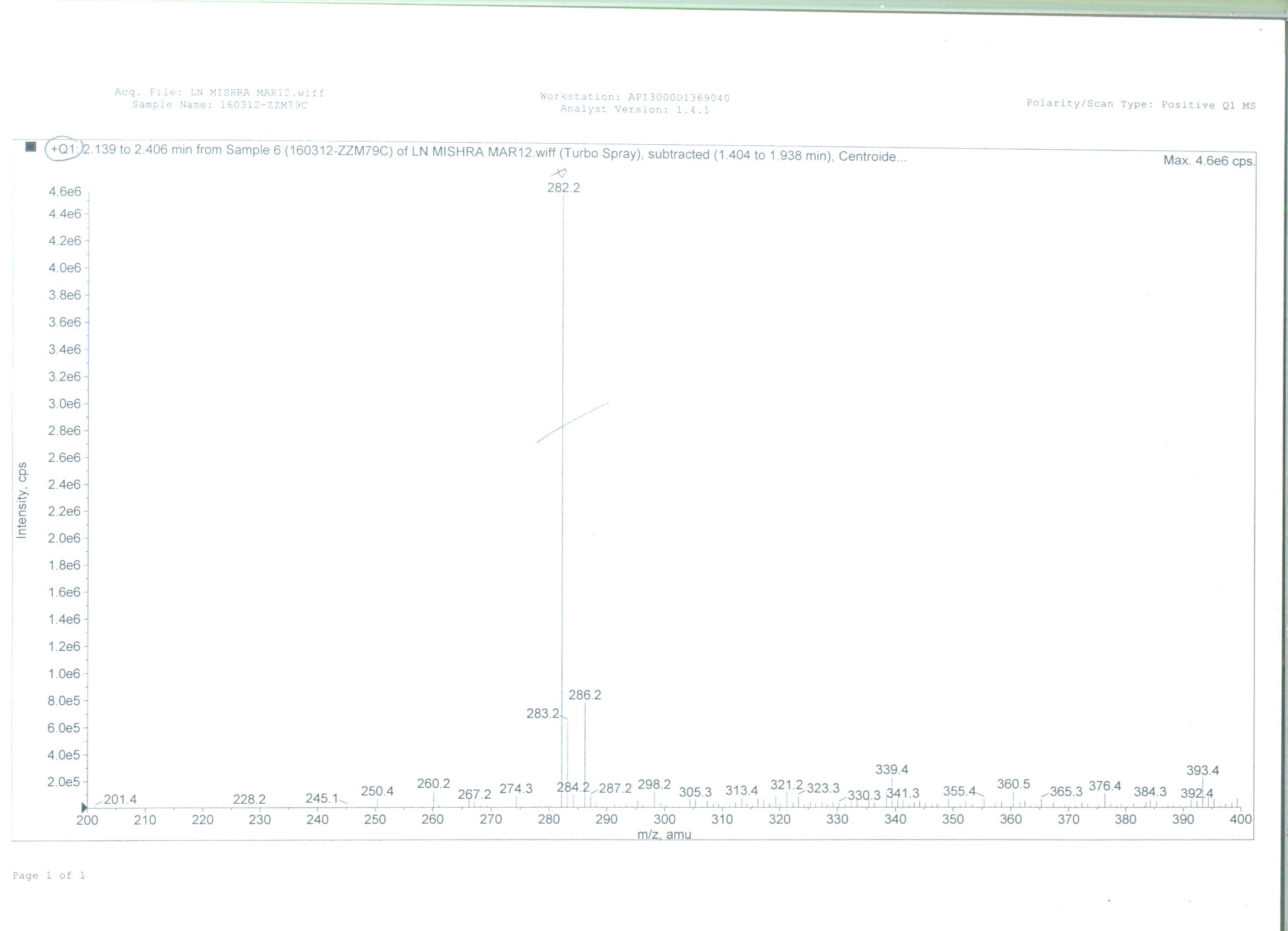
**

**Figure 3d:** Mass of compound **3**

**
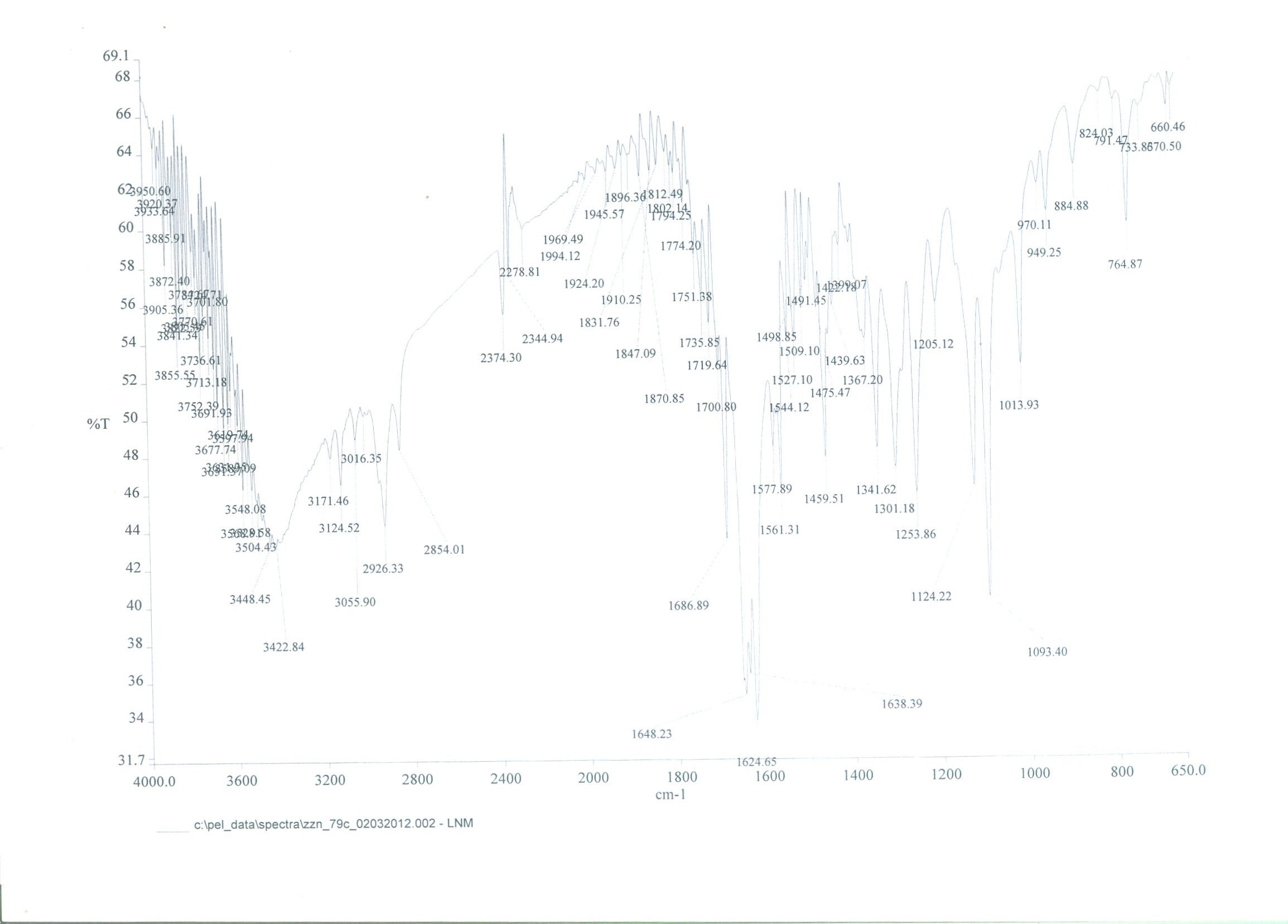
**

**Figure 3e**: IR of compound **3**

**
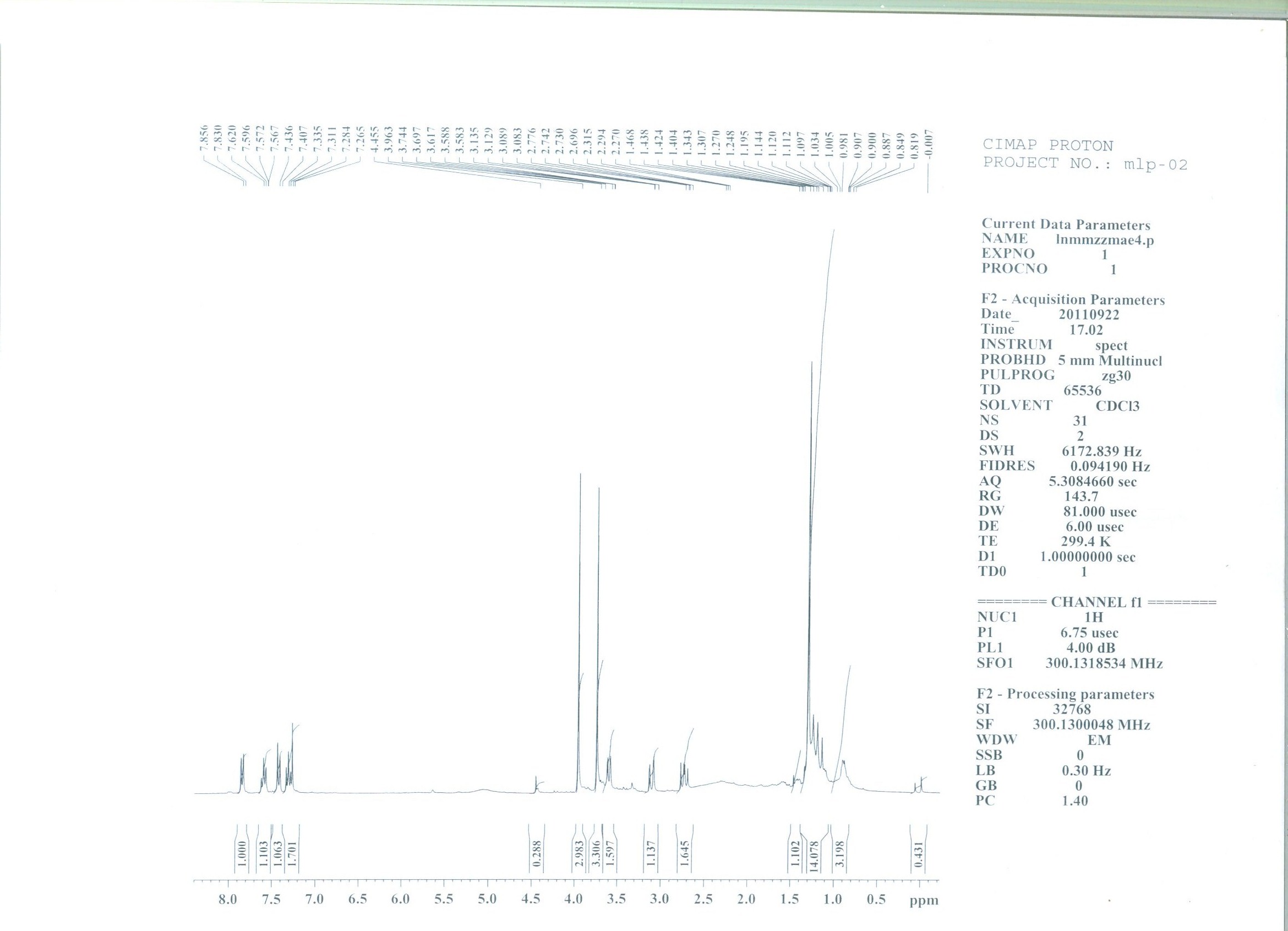
**

**Figure 4a**: ^1^H NMR of compound **8**

**
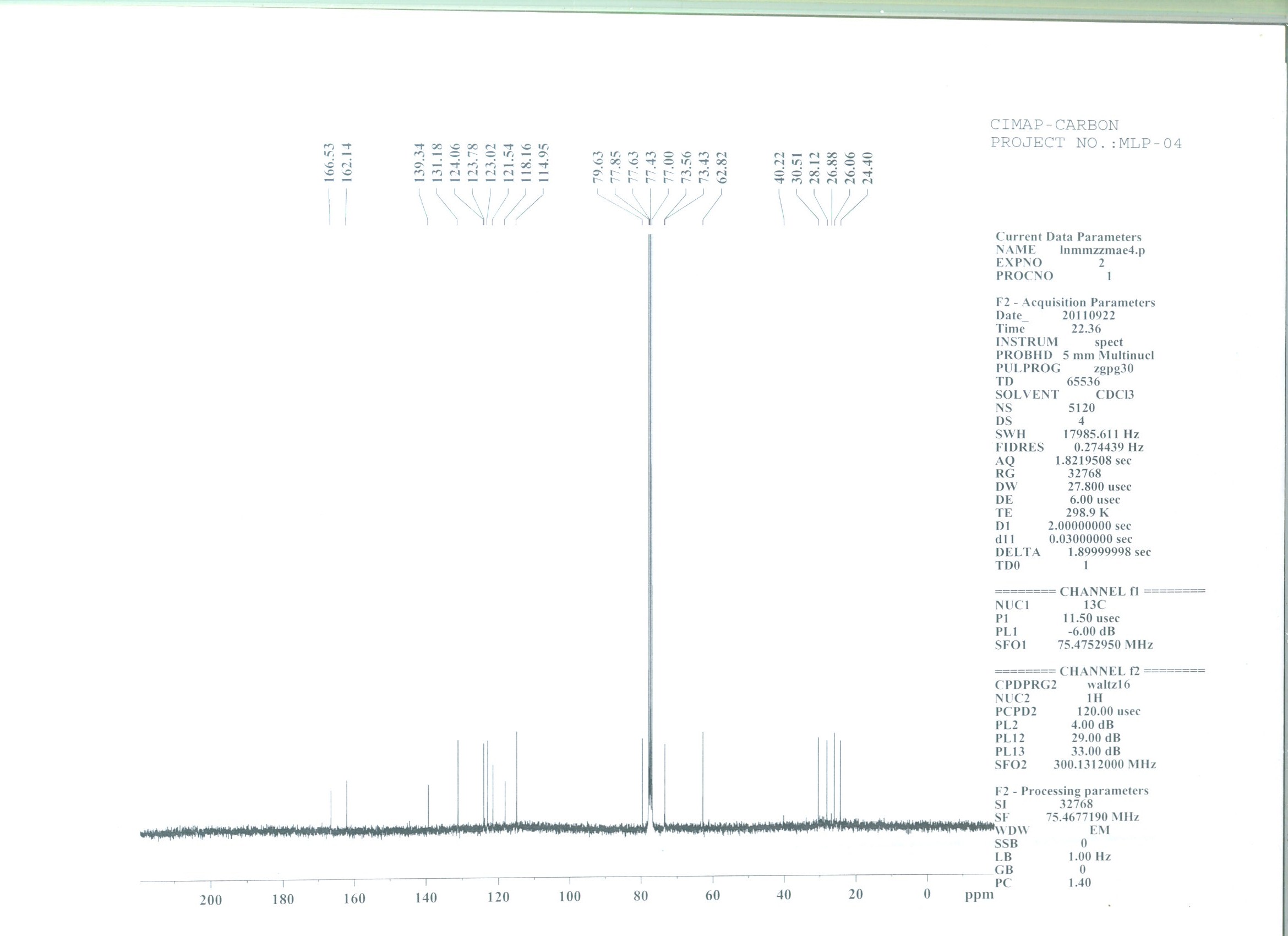
**

**Figure 4b:** ^13^C NMR of compound **8**

**
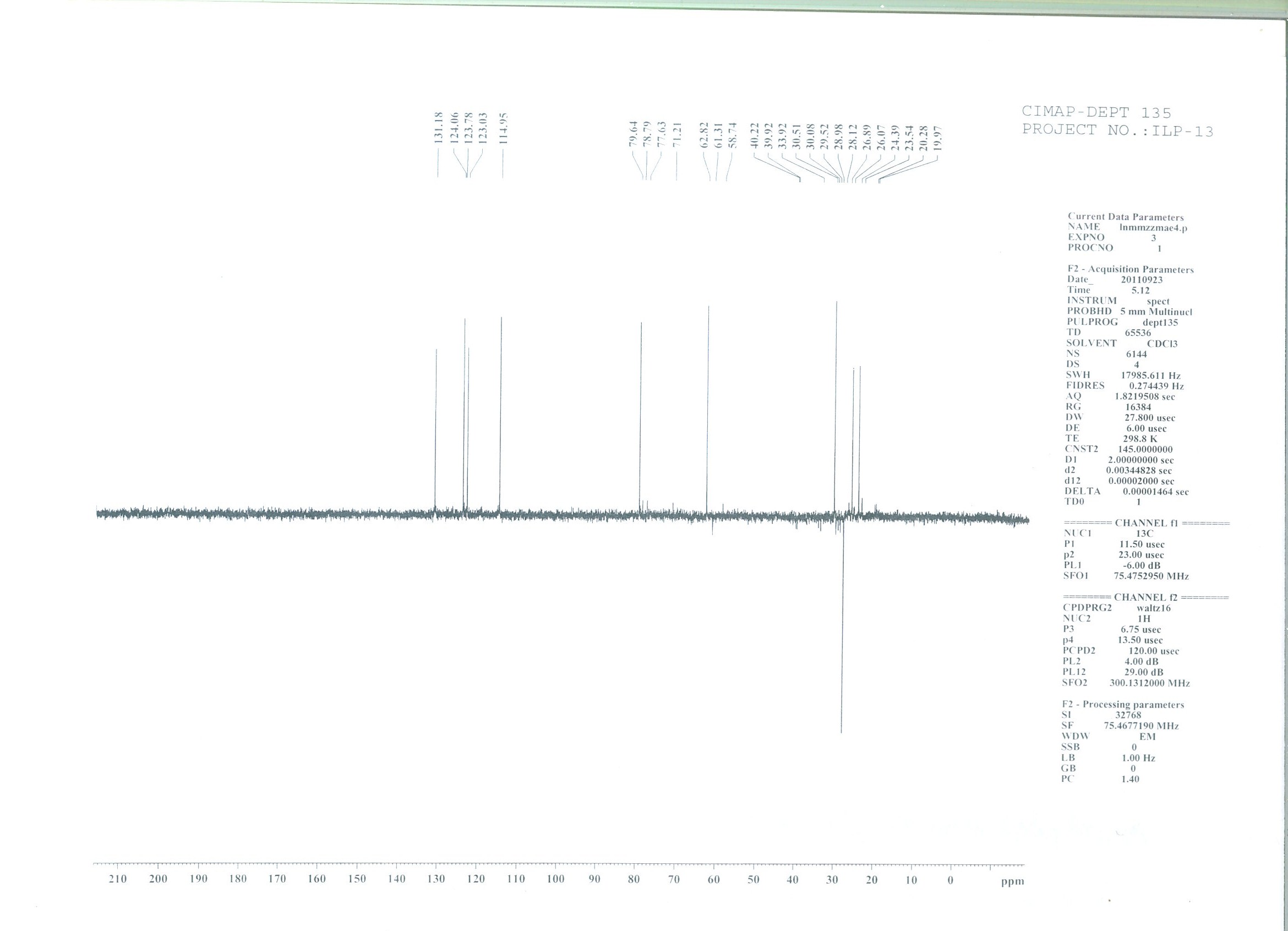
**

**Figure 4c:** DEPT of compound **8**

**
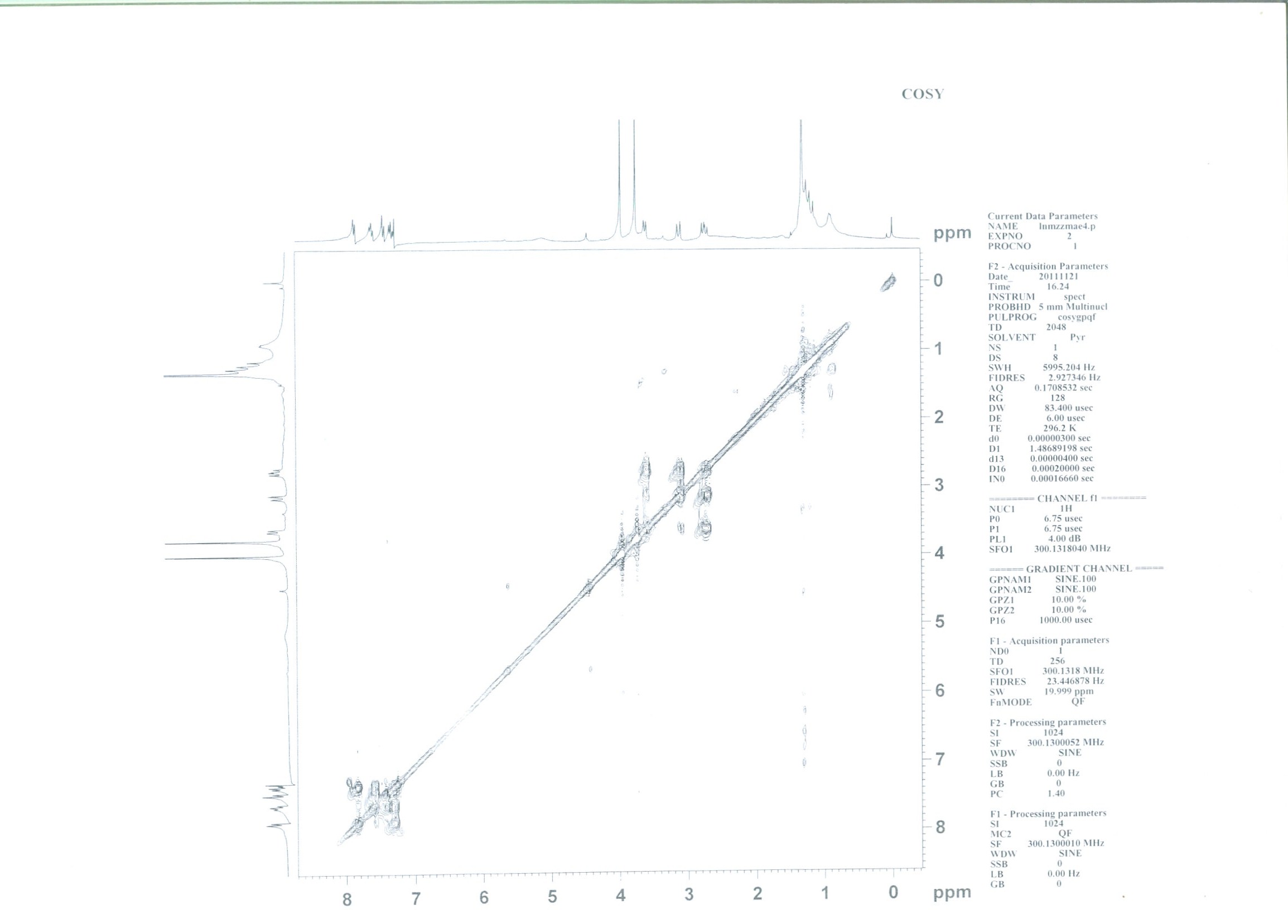
**

**Figure 4d:** COSY of compound **8**

**
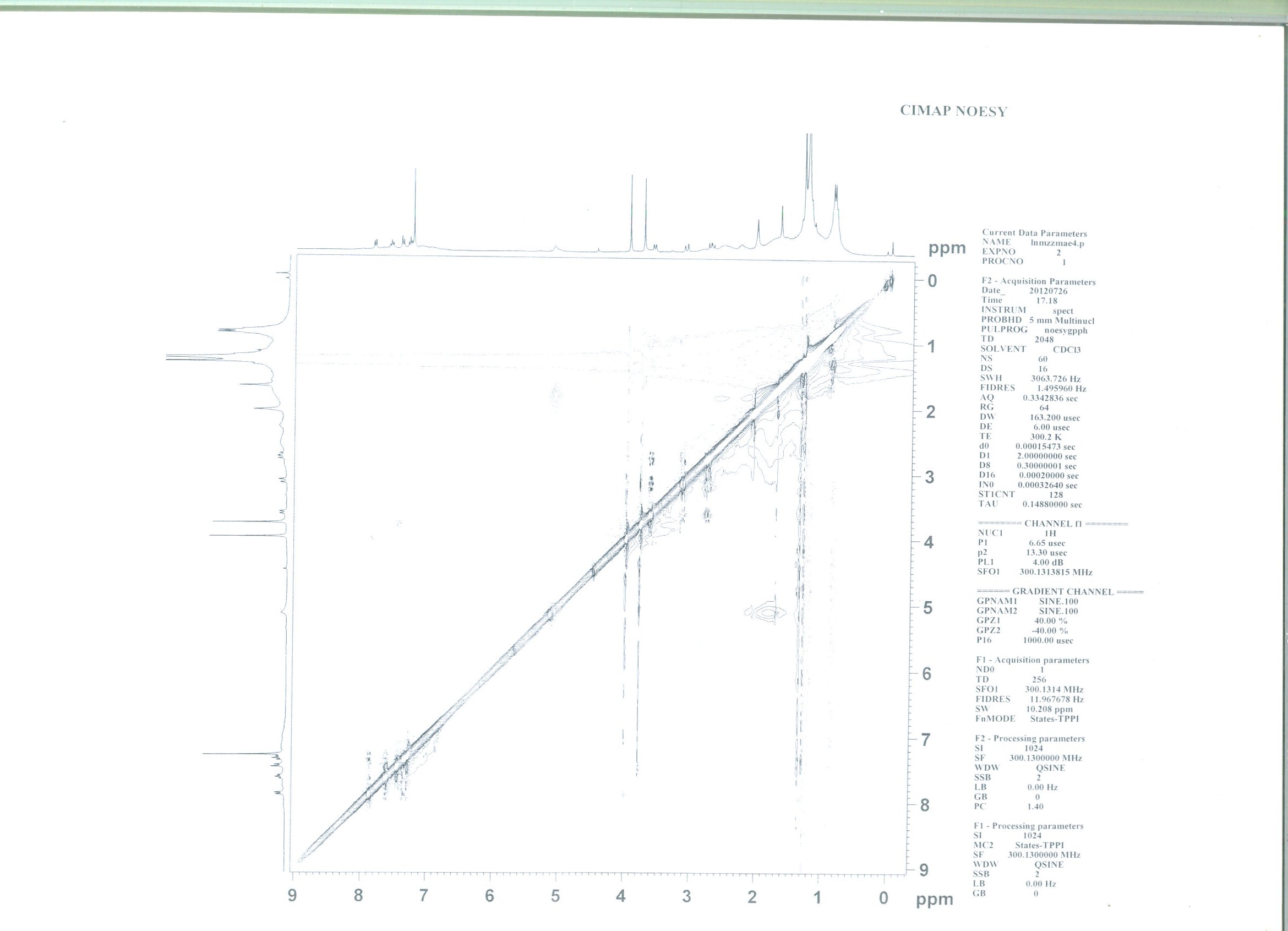
**

**Figure 4e:** NOESY of compound **8**

**
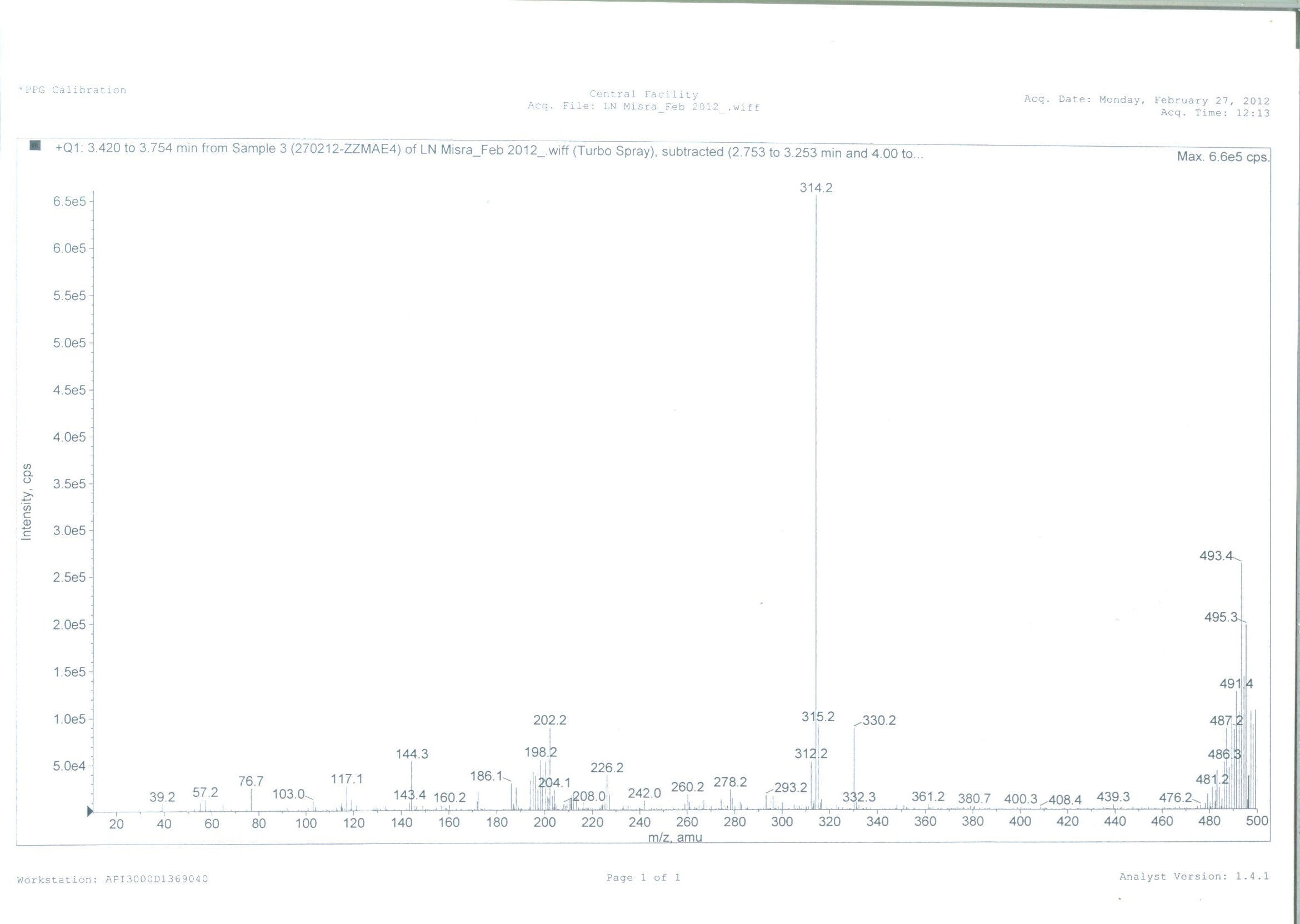
**

**Figure 4f:** Mass of compound **8**

**
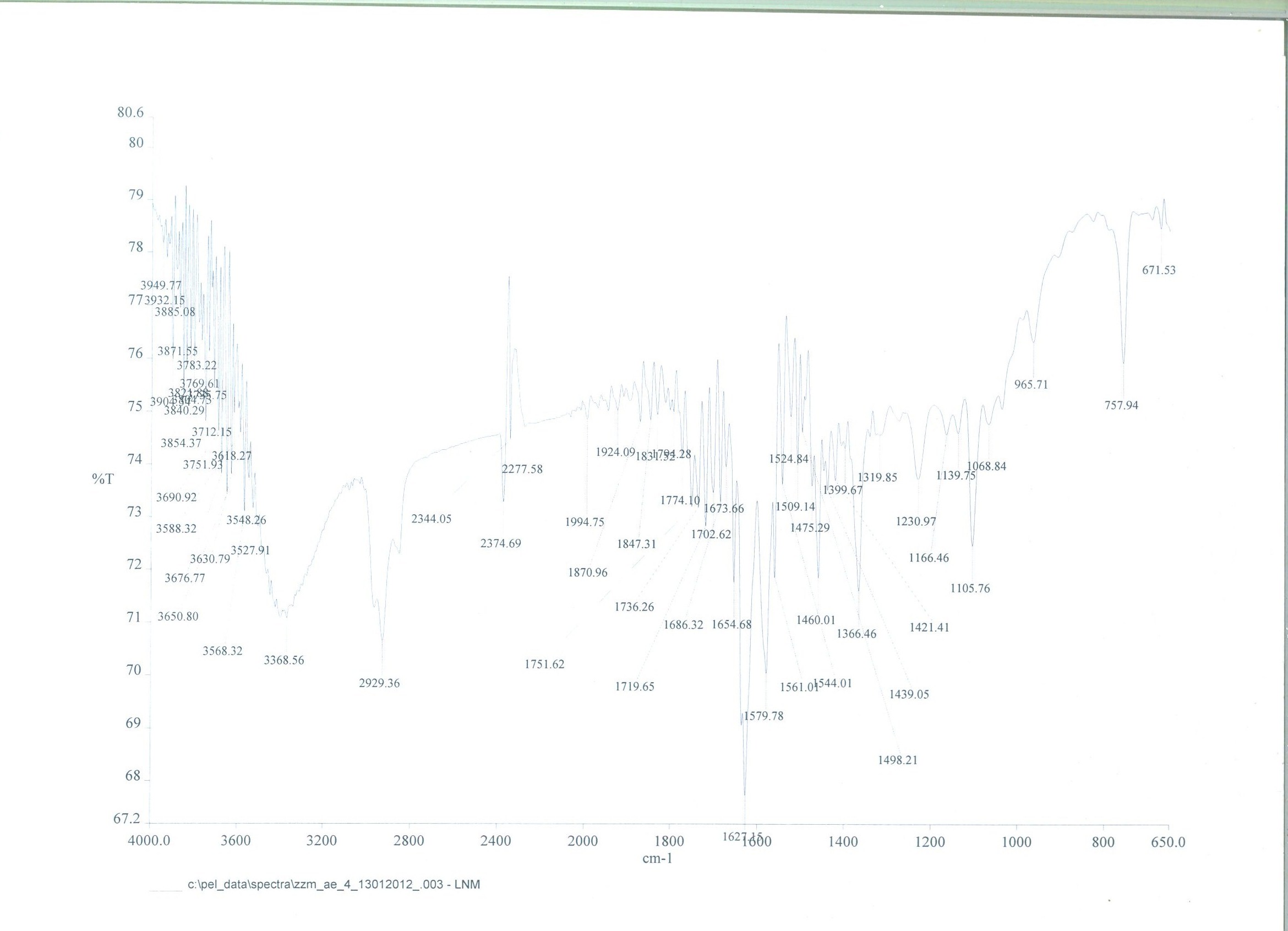
**

**Figure 4g:** IR of compound **8**

**
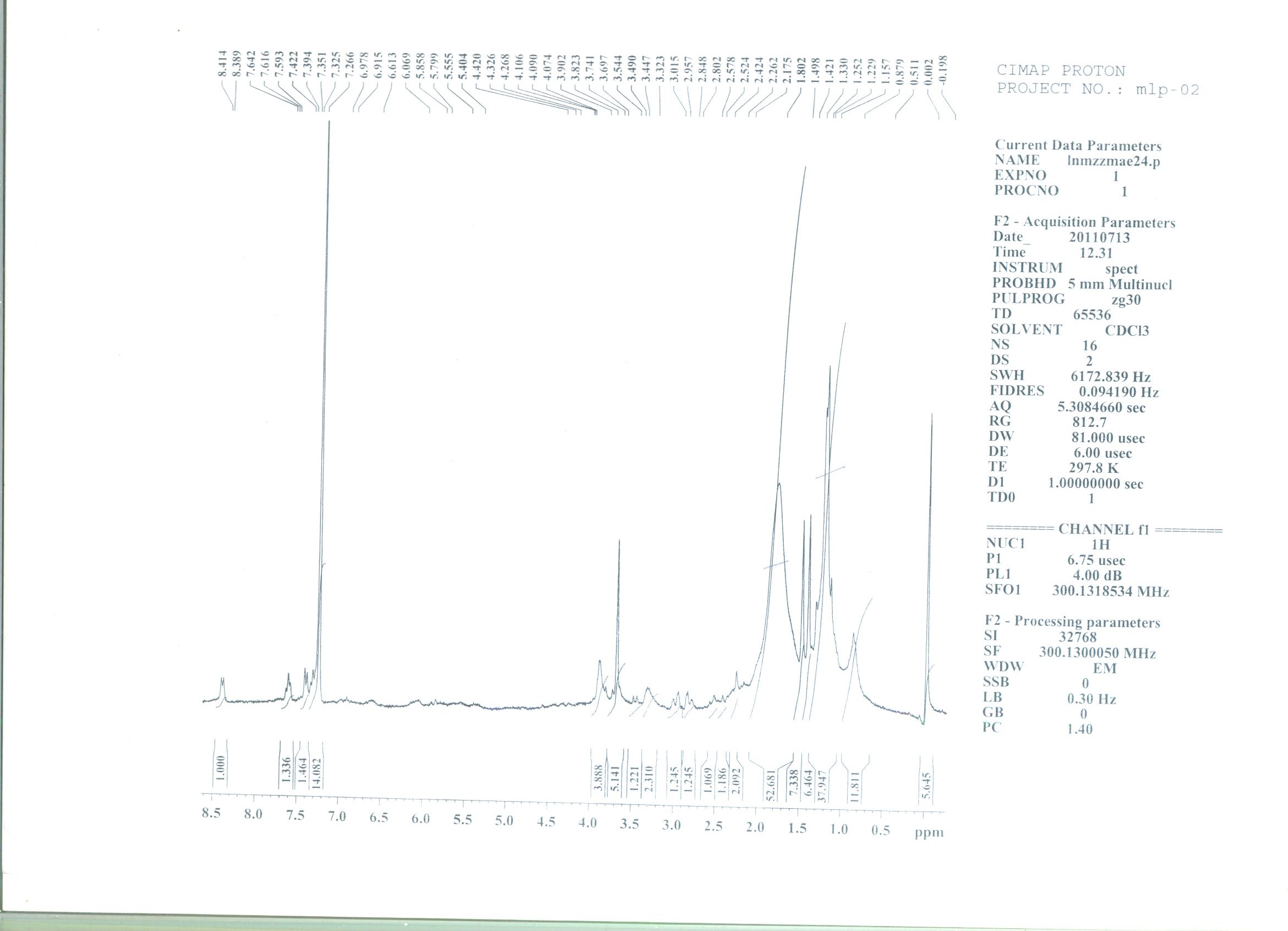
**

**Figure 5a:** ^1^H NMR of compound **9**

**
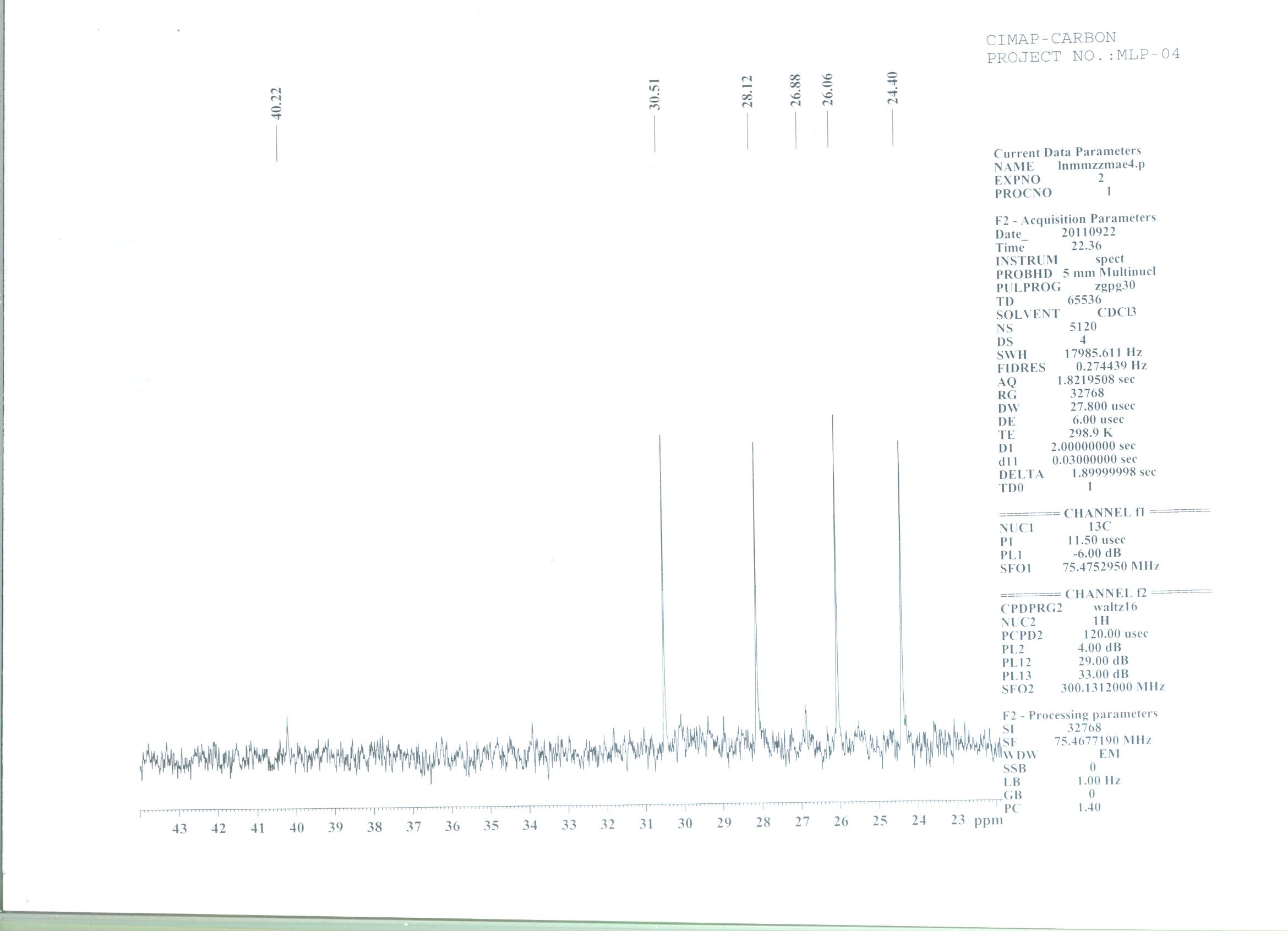
**

**Figure 5b:** ^13^C NMR of compound **9**

**
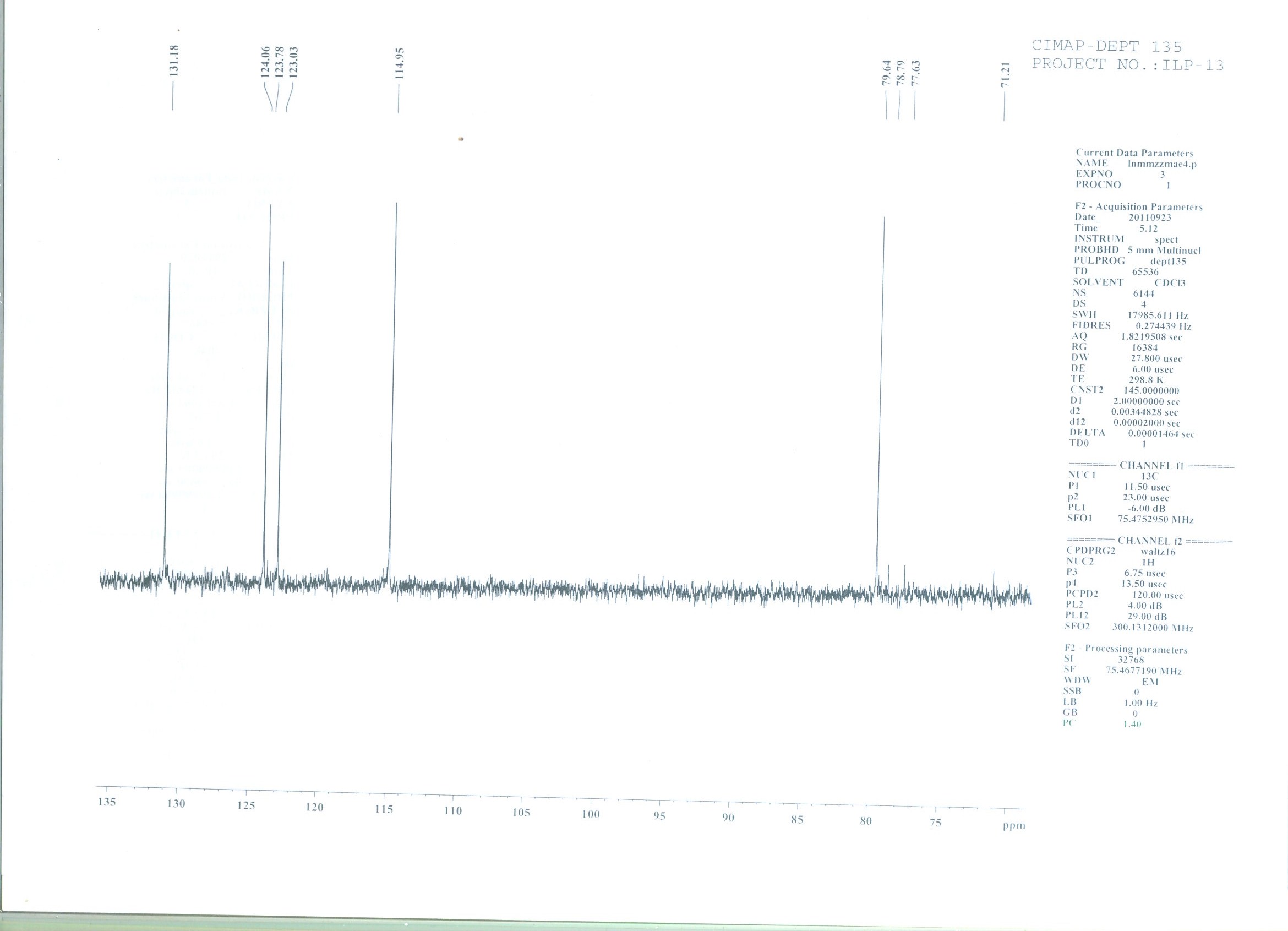
**

**Figure 5c:** ^13^C NMR of compound **9**

**
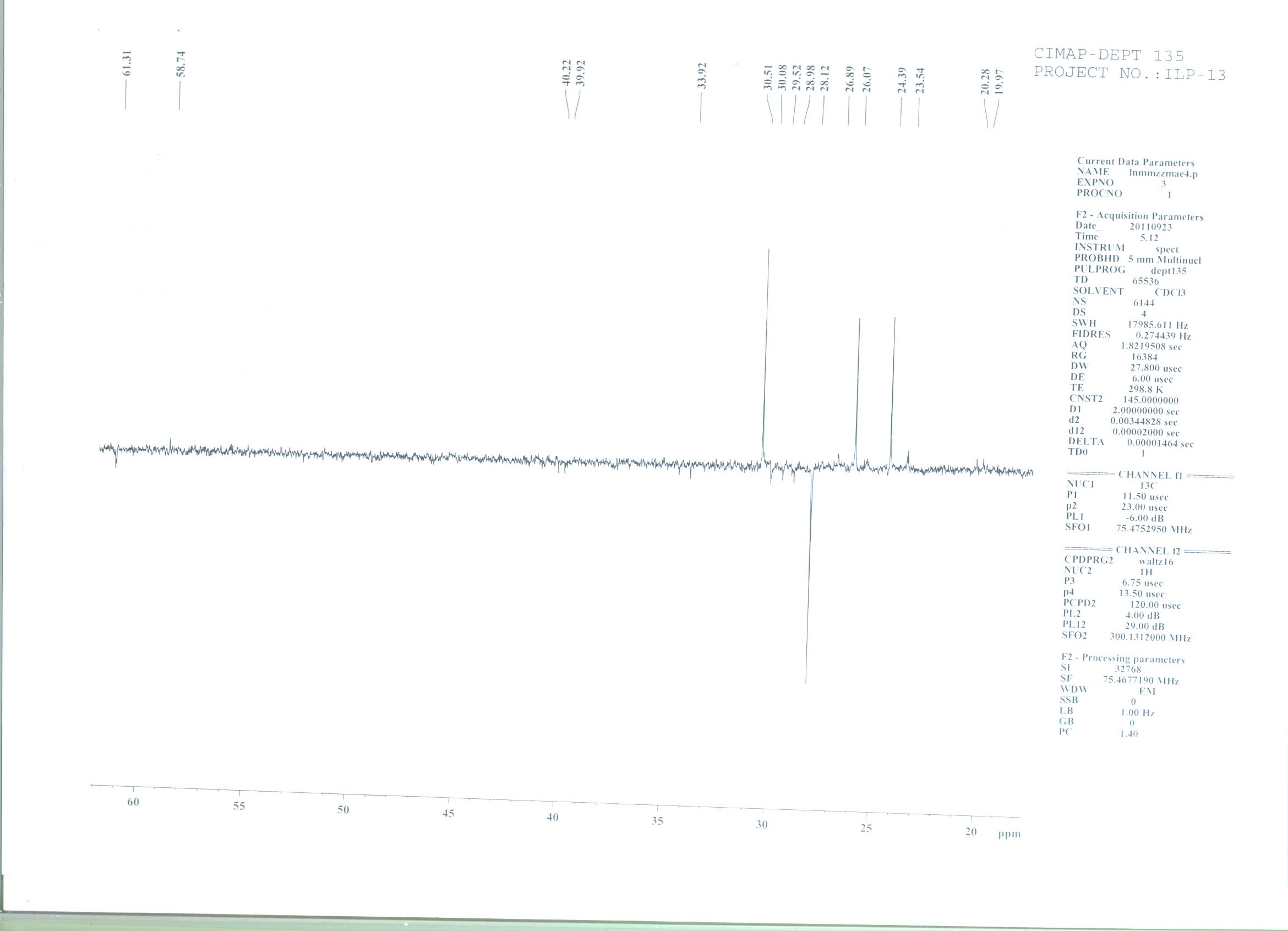
**

**Figure 5d:** DEPT of compound **9**

**
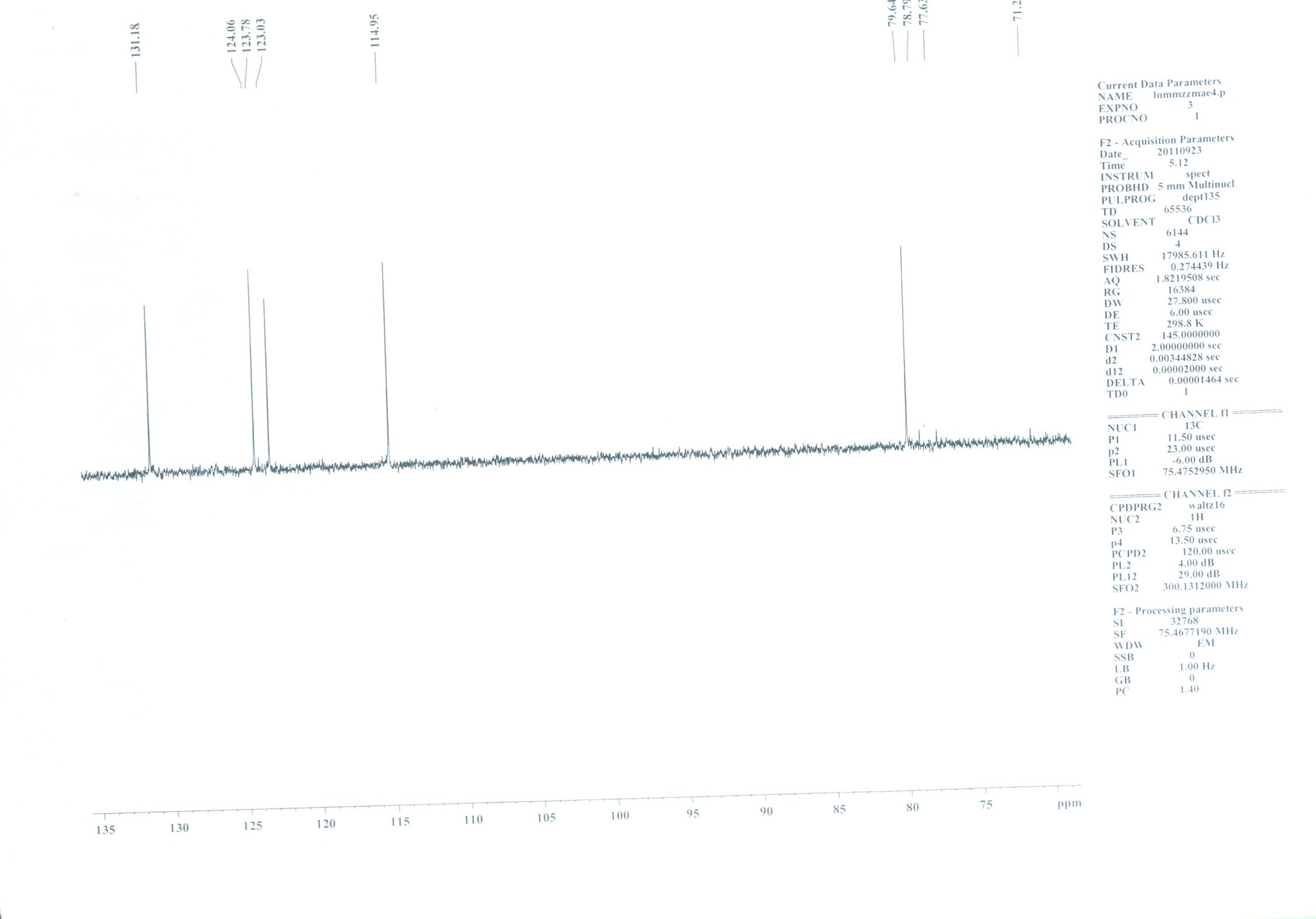
**

**Figure 5e:** DEPT of compound **9**

**
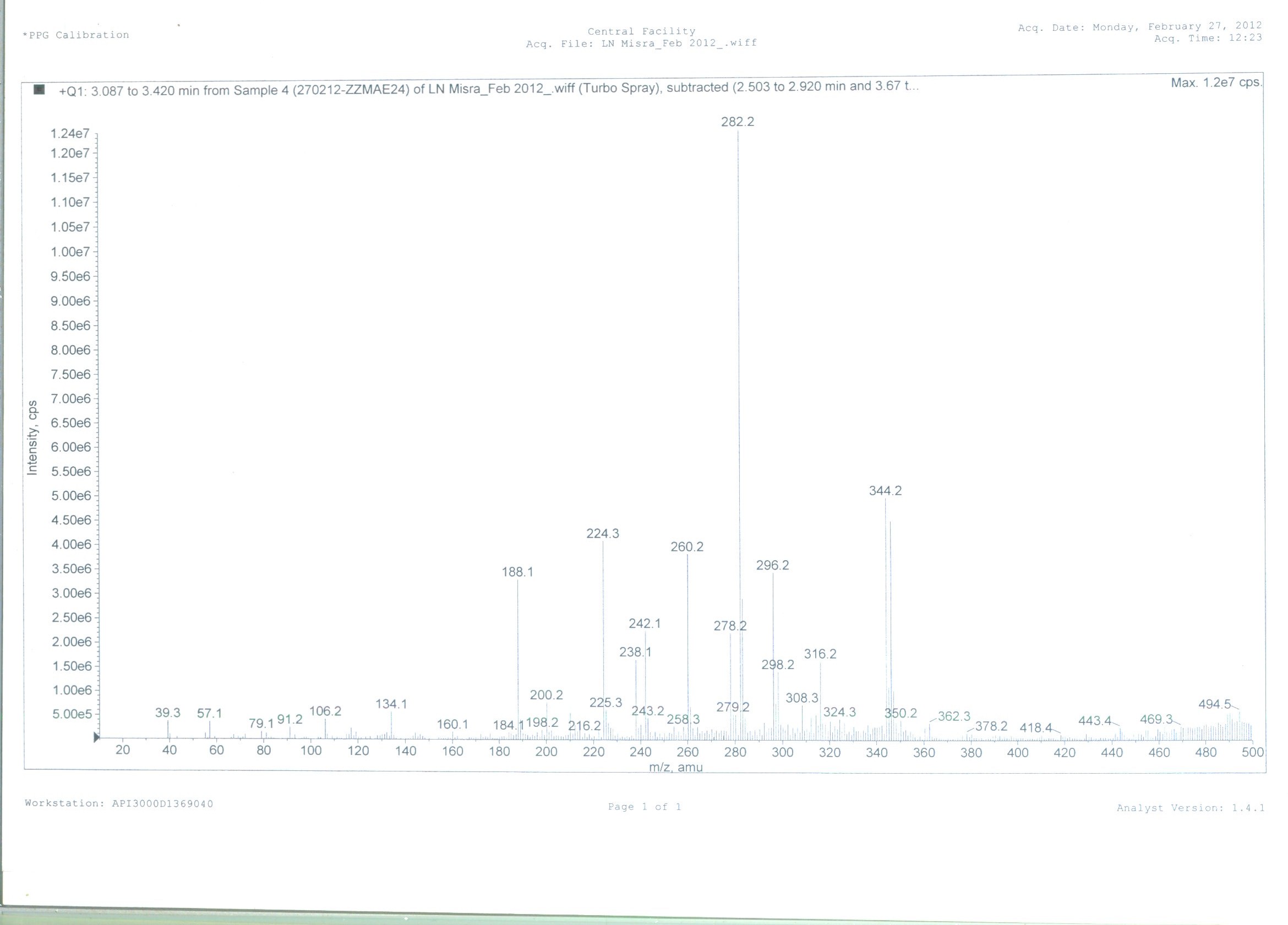
**

**Figure 5f:** Mass of compound **9**

**
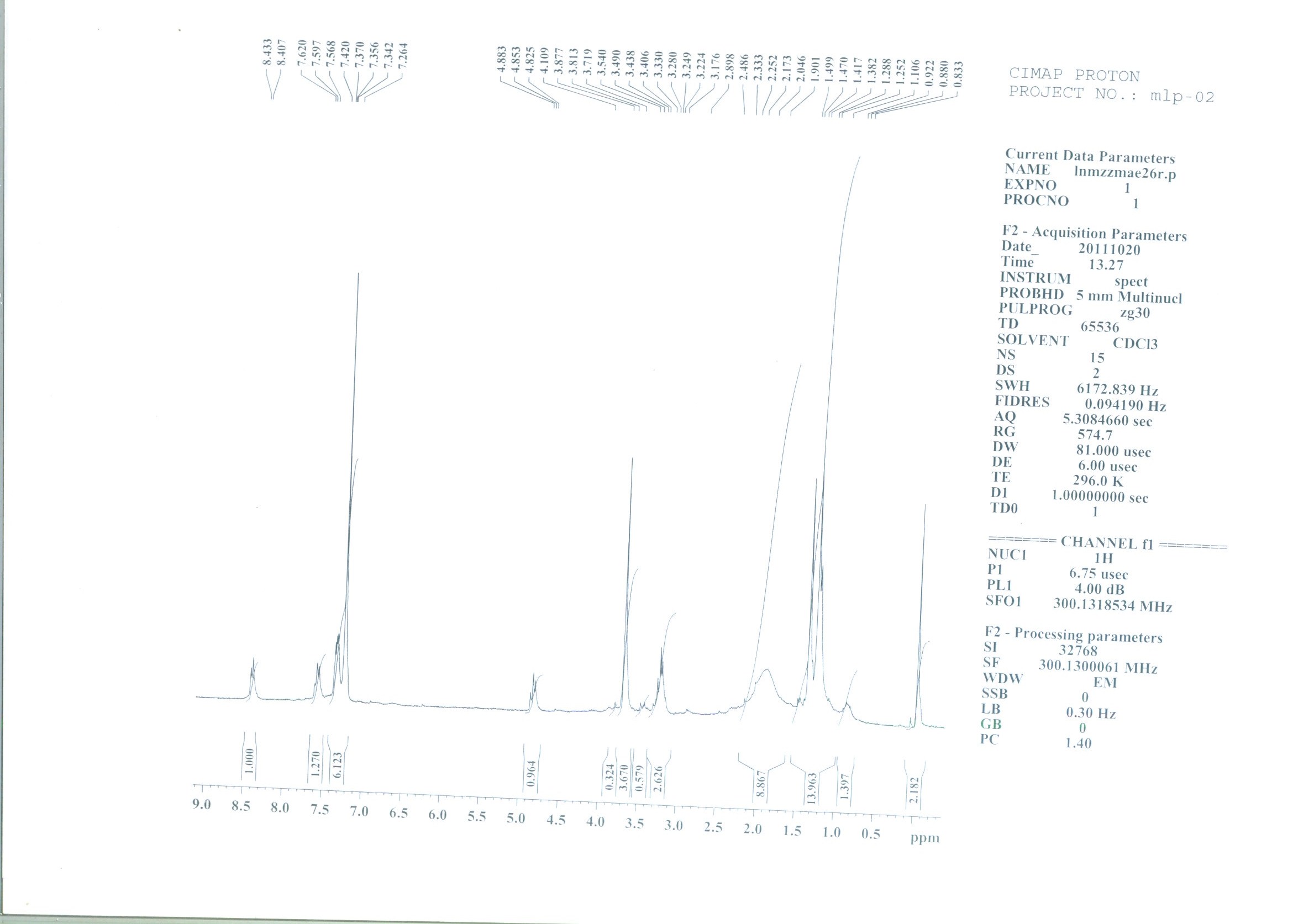
**

**Figure 6a:** ^1^H NMR of compound **10**

**
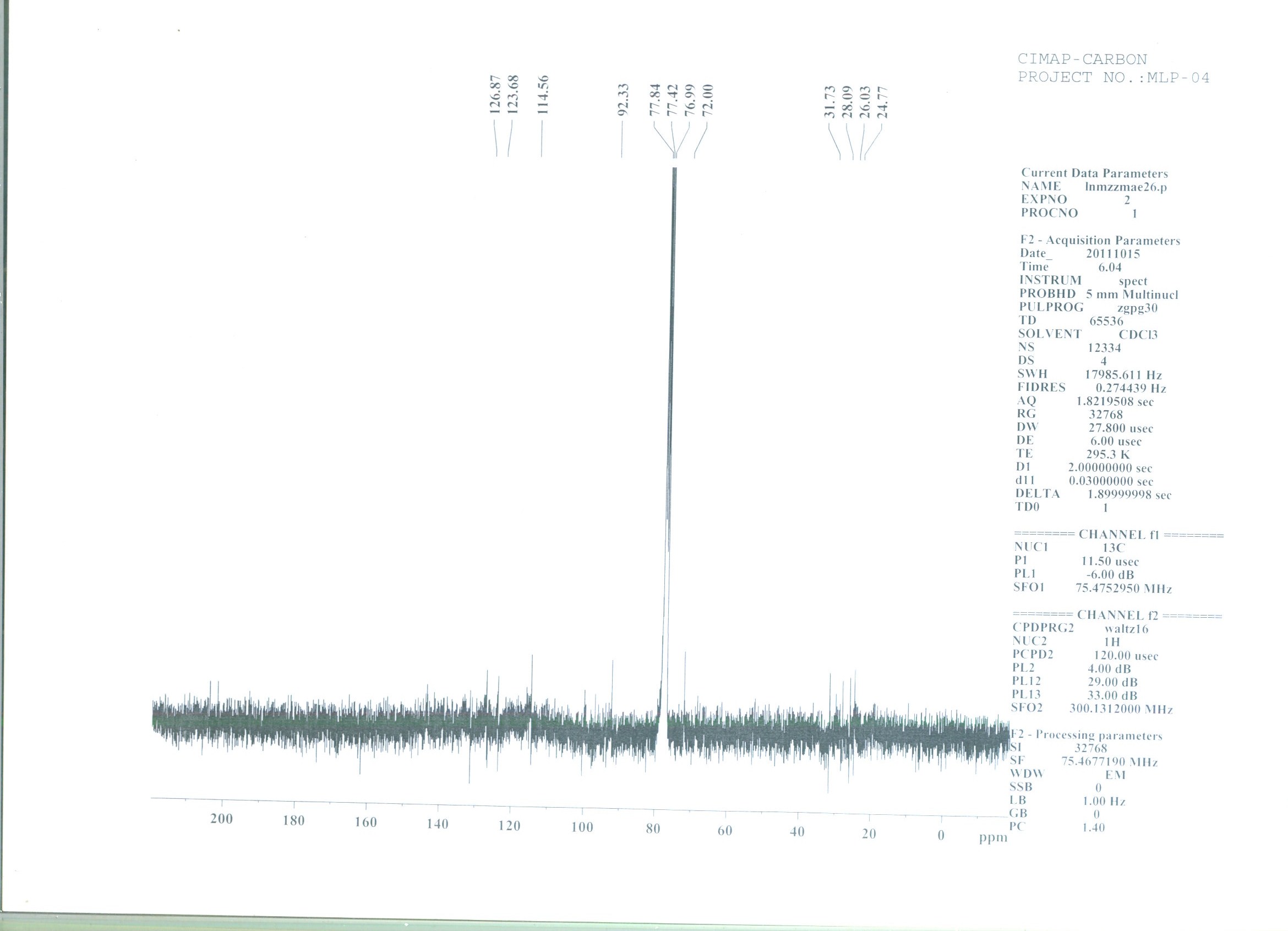
**

**Figure 6b:** ^13^C NMR of compound **10**

**
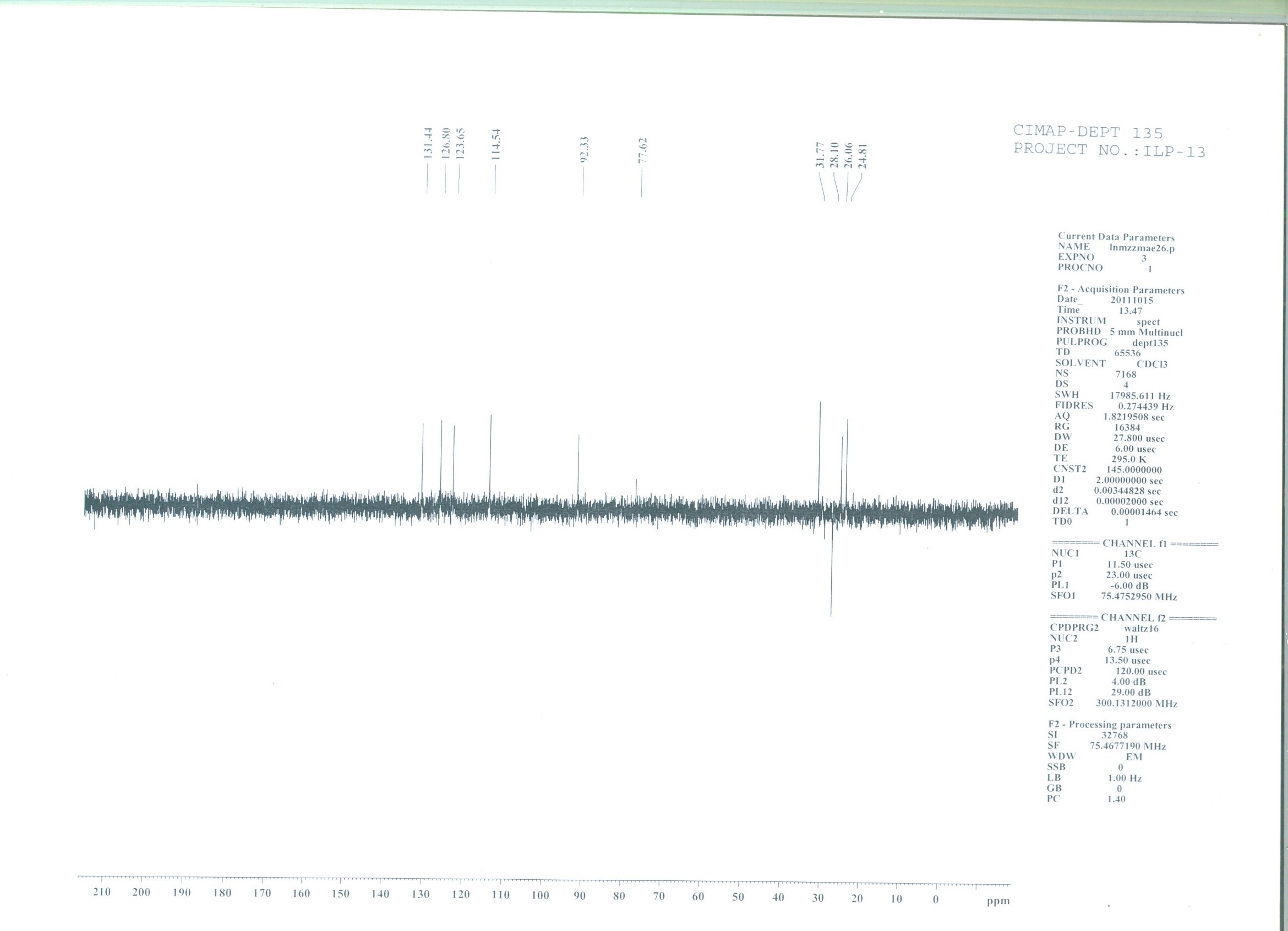
**

**Figure 6c:** DEPT of compound **10**

**
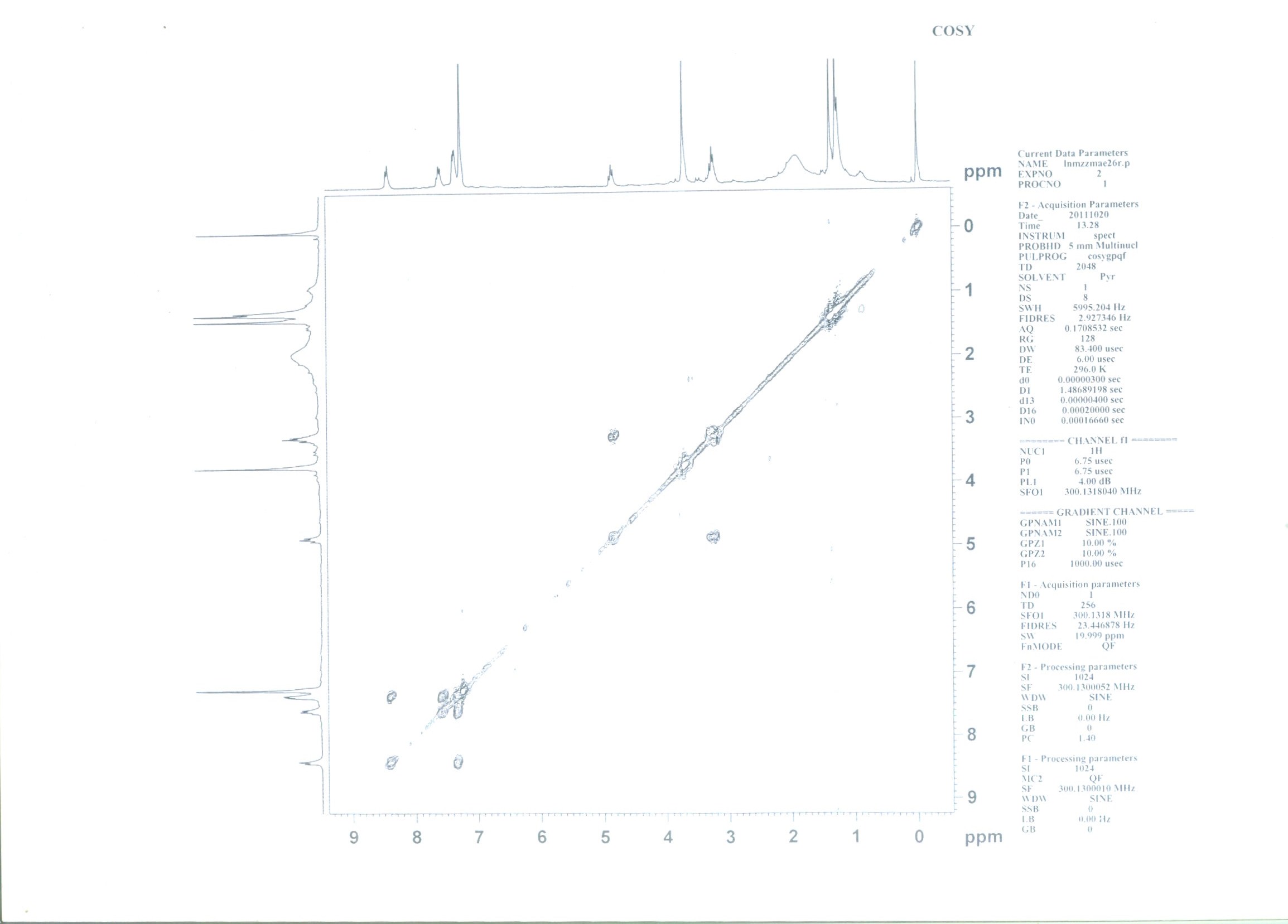
**

**Figure 6d:** COSY of compound **10**

**
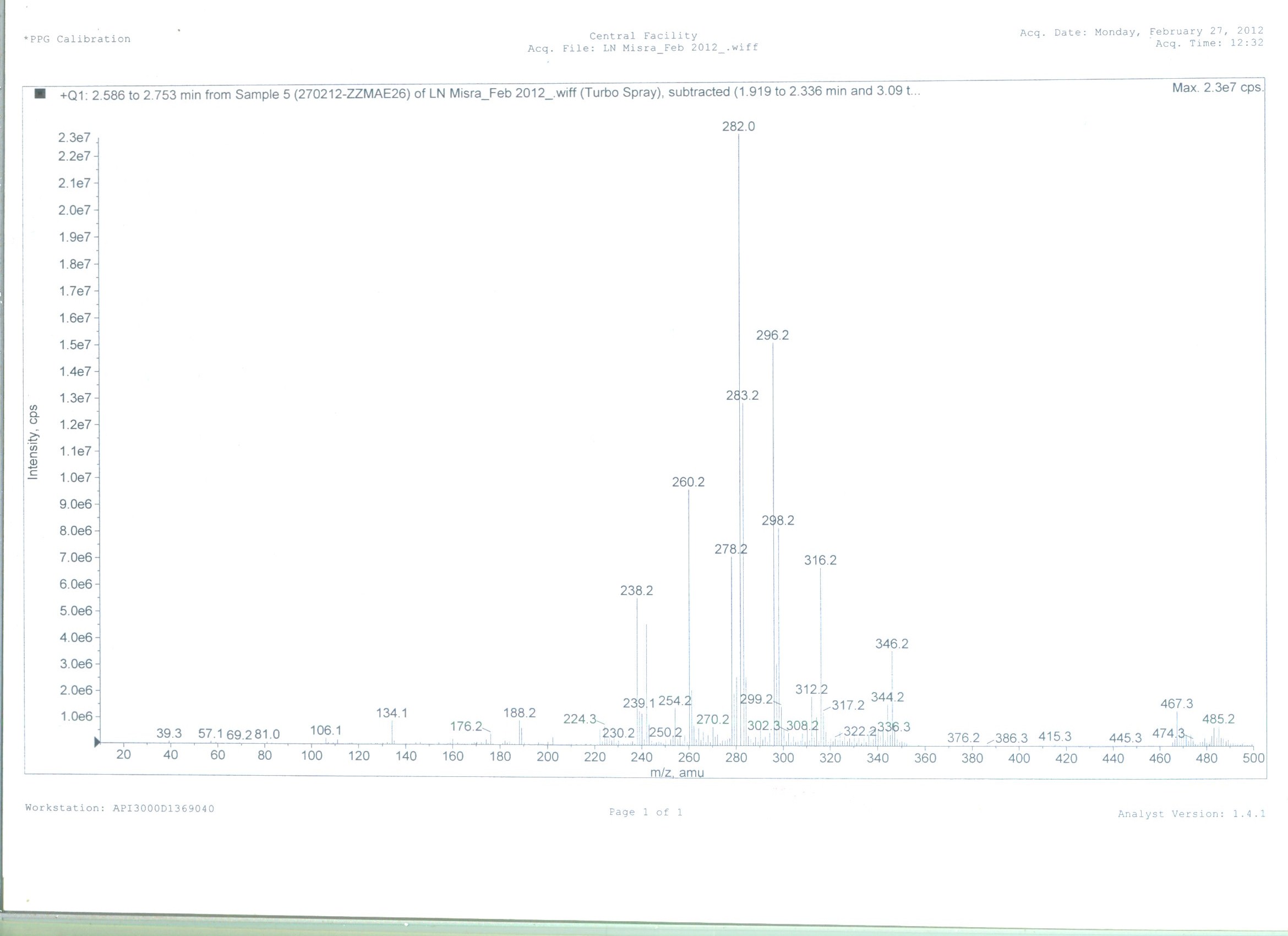
**

**Figure 6e:** Mass of compound **10**

**
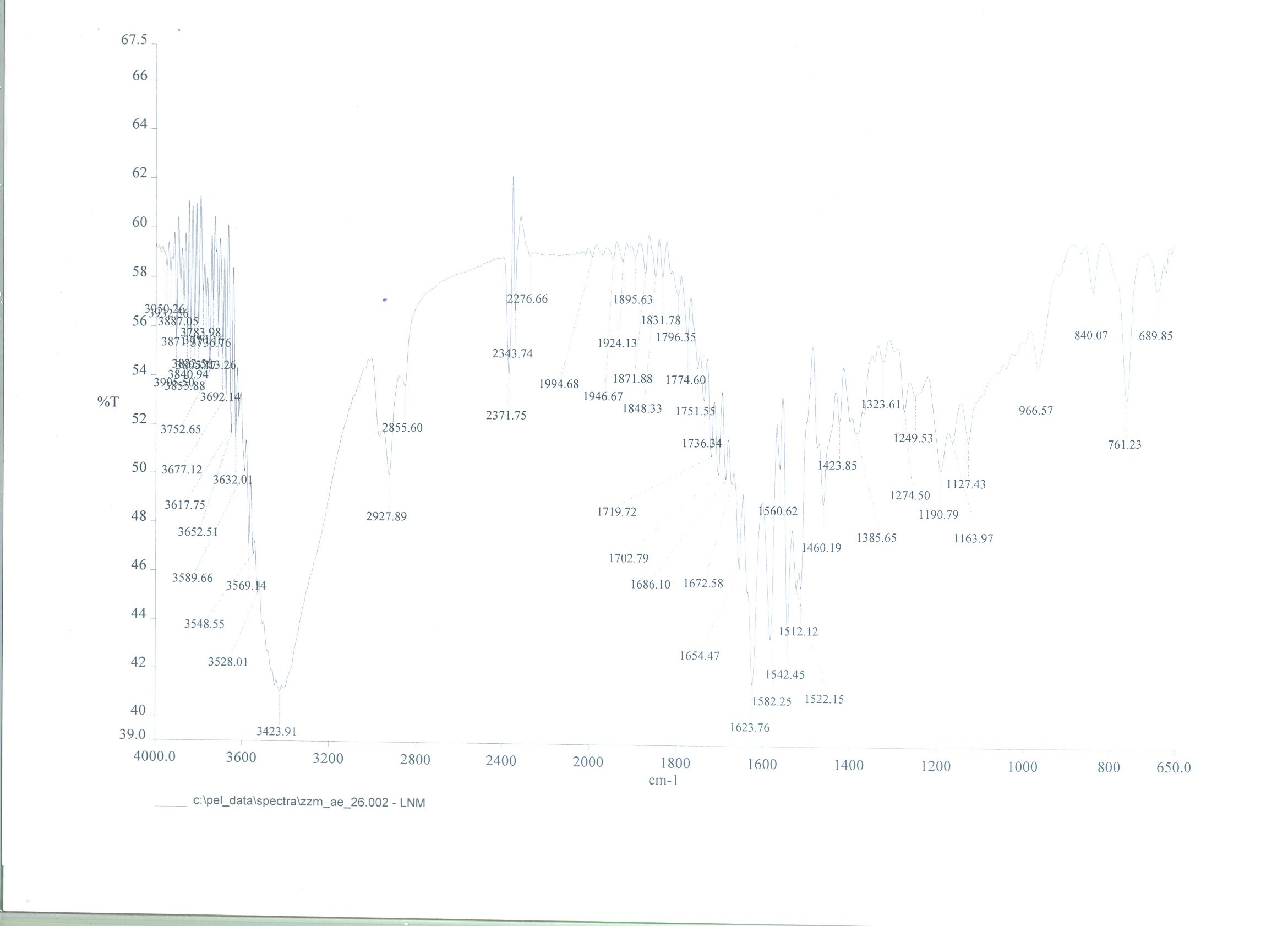
**

**Figure 6f:** IR of compound **10**
